# Influence of LED spectral quality and natural growing conditions on flowering, yield, and fruit biochemical quality of okra (*Abelmoschus esculentus* (L.) Moench)

**DOI:** 10.3389/fpls.2026.1947730

**Published:** 2026-10-29

**Authors:** Yao Hervé Yao, Banah Florent Degni, Laurent Canale, Arouna Khalil Fanny, Cissé Théodore Haba, Georges Zissis

**Affiliations:** 1 Laboratoire d’Ingénierie Électronique, d’Électricité et des Systèmes Embarqués (LIEESE), Institut National Polytechnique Félix Houphouët Boigny (INP-HB), Yamoussoukro, Côte d’Ivoire; 2 University of Toulouse, Toulouse INP, CNRS, Laboratoire Plasma et Conversion d’Énergie (LAPLACE), Toulouse, France; 3 French National Center of Scientific Research (CNRS), Laboratoire Plasma et Conversion d’Énergie (LAPLACE), Unité Mixte de Recherche (UMR) 5213, Toulouse, France

**Keywords:** antioxidant activity, climate change, controlled-environment agriculture, crop yield, light-emitting diodes (LEDs), light spectra, okra, plant growth

## Abstract

**Introduction:**

Climate change poses significant challenges to open-field production of okra (*Abelmoschus esculentus* (L.) Moench), a tropical vegetable crop of high nutritional and economic value. This study evaluated the effects of light-emitting diode (LED) spectral quality on reproductive development, fruit characteristics, yield, mineral composition, antioxidant activity, and total phenolic content of okra.

**Methods:**

Plants were grown under blue (452 nm), red (659 nm), and combined red/blue (9:1; 657/447 nm) LED lighting at a photosynthetic photon flux density (PPFD) of 400 µmol m^−2^ s^−1^ under a 10-h photoperiod and compared with greenhouse and outdoor conditions.

**Results:**

The red/blue 9:1 (RB 9:1) treatment promoted earlier flowering, with anthesis occurring approximately 37 days after sowing, and showed the highest numerical fruit length and fresh fruit mass, as well as the highest yield per plant and estimated yield per hectare, the latter being approximately twice that of the other growing conditions. RB 9:1 showed the highest numerical concentrations of calcium, potassium, sulfur, copper, and zinc, while outdoor plants showed the highest numerical concentrations of iron and strontium and red light the highest numerical rubidium concentration. RB 9:1 showed the highest numerical antioxidant activity, although it was not significantly different from red or blue light. Blue light showed the highest numerical total phenolic content, with no significant differences among treatments.

**Discussion:**

Overall, RB 9:1 showed promising potential for earlier reproductive development and fruit production under the tested conditions.

## 1 Introduction

Flowering is a key developmental stage in plants resulting from the integration of environmental and endogenous signals, among which photoperiod plays a central role (SharathKumar et al., 2024). Plants precisely adjust the timing of the floral transition to ensure reproductive success and are traditionally classified, according to their photoperiodic response, as short-day (SD), long-day (LD), or day-neutral species (SharathKumar et al., 2021). The photoperiodic response primarily depends on the duration of the dark period, referred to as the scotoperiod. The external coincidence model describes how the timing of external light signals is integrated with the plant’s internal circadian clock to regulate light-dependent molecular and physiological responses. In short-day plants, flowering is induced when the scotoperiod exceeds a critical threshold, whereas in long-day plants flowering is promoted when the dark period is shorter, in accordance with the external coincidence model (Song et al., 2015). At the molecular level, photoperiodic flowering is regulated through the perception of light signals that synchronize the circadian clock, a key component of the plant timekeeping system responsible for measuring day length. According to the external coincidence model, the interaction between the circadian clock and light controls floral induction (Johansson and Staiger, 2015). Under inductive photoperiods, this regulation is achieved through the activation of the florigen gene *FLOWERING LOCUS T (FT)* and the repression of anti-florigenic factors such as *ANTI-FLORIGENIC FT (AFT)* and *TERMINAL FLOWER 1 (TFL1)*, a regulatory mechanism conserved in both long-day and short-day plant species (Higuchi, 2018).

Beyond photoperiod, light spectral composition is another major environmental factor regulating flowering in both short-day and long-day plants (Cerdán and Chory, 2003; Song et al., 2015). Red light has been widely reported to effectively inhibit flowering in short-day species (SharathKumar et al., 2021). However, the response to spectral quality is highly species-dependent. For example, in chrysanthemum, a short-day species, red and blue light play distinct yet complementary roles. Under short-day conditions, both wavelengths independently induce flowering, whereas their simultaneous combination accelerates flowering compared with monochromatic light treatments (Higuchi et al., 2012). By contrast, in Kalanchoe, a qualitative short-day plant, red light induces earlier flowering than blue light under short-day conditions (Amaki and Kunii, 2015). These findings demonstrate that the effects of light spectral quality on flowering in short-day plants are strongly species-specific, highlighting the complexity of photoperiodic regulation and the need for species-oriented approaches. In horticultural production systems, artificial lighting is widely used to enhance plant growth and development, particularly by extending the photoperiod. However, this strategy is not well suited to short-day plants, which require a minimum uninterrupted dark period to initiate flowering. Consequently, in these species, the photoperiod should generally not exceed 12–14 h to maintain floral induction (SharathKumar et al., 2021). Nevertheless, LED technology enables the implementation of dynamic lighting strategies, in which the light spectrum and/or light intensity can be adjusted according to the time of day or the developmental stage of the crop (SharathKumar et al., 2020).

With respect to okra (*A. esculentus* L.), a facultative (quantitative) short-day species belonging to the Malvaceae family, relatively few studies have investigated the regulation of flowering under LED lighting. Hamamoto and Yamazaki demonstrated that extending the photoperiod or providing night-break lighting significantly delayed flowering in okra, with the strongest inhibitory effect observed under red light (Hamamoto and Yamazaki, 2009). Although roselle, another member of the Malvaceae family, also appears to perceive red light, its reproductive response is considerably weaker than that of okra cultivars, and there is little evidence of marked sensitivity to blue or green light (Hamamoto and Yamazaki, 2009). More recently, a 14-h photoperiod applied for 30 days under LED lighting was shown to delay flowering compared with natural growing conditions characterized by a 12-h photoperiod (Yao et al., 2025). Under short-day conditions, red light induced earlier flowering than blue light under LED lighting (Yao et al., 2025). Collectively, these findings indicate that the mechanisms underlying the regulation of flowering in okra in response to photoperiod and light spectral quality remain only partially understood and warrant further investigation. Okra is an economically important vegetable crop and ranks second only to tomato among commonly consumed vegetables in West Africa, with global production reaching approximately 11.2 million tons in 2023 (FAOSTAT, 2023; Hamon, 1987). Previous studies have demonstrated that a 10-h photoperiod effectively promotes flowering, anthesis, and fruit quality in okra (Maduagwuna et al., 2019; Nwoke, 1983). However, these studies were conducted under natural environmental conditions. In controlled-environment horticulture, energy consumption is an important consideration in the design and operation of artificial lighting systems. Shorter photoperiods may potentially reduce lighting demand; however, the associated energy and economic benefits depend on several factors, including lighting-system efficiency, electricity costs, and crop productivity. Therefore, any potential energy-saving benefits associated with shorter photoperiods require specific evaluation. Therefore, it is essential to investigate flowering responses of okra under artificial LED lighting using a 10-h photoperiod and to compare these responses with those observed under natural growing conditions.

In addition to flowering, light also influences the nutritional quality of fruits and vegetables (Landi et al., 2020). Simultaneous exposure to red and blue light is generally considered more effective than monochromatic lighting in enhancing plant growth and nutritional quality (Chen and Yang, 2018; Ma et al., 2018). Plant nutritional quality largely depends on the accumulation of essential macro- and micronutrients, including nitrogen, phosphorus, potassium, magnesium, calcium, iron, zinc, manganese, and copper (Bouain et al., 2019). Variations in the proportions of red and blue light have been shown to markedly affect the uptake and accumulation of these mineral elements in crops such as lettuce, basil, celery, and mulberry (Liu et al., 2025; Pennisi et al., 2019, 2019). Moreover, monochromatic red light has been reported to promote the accumulation of certain macronutrients, particularly nitrogen and magnesium, in lettuce (Amoozgar et al., 2017). In okra, Degni et al (Degni et al., 2021), reported that the highest concentrations of calcium, phosphorus, and manganese were obtained under red light, whereas a more recent study showed that blue light enhanced sulfur and manganese accumulation more effectively than either red light or alternating red and blue light treatments (Degni et al., 2025). These light spectra have also been shown to enhance antioxidant activity and total phenolic content in amaranth microgreens and lamb’s lettuce (Długosz-Grochowska et al., 2016; Toscano et al., 2021). However, to the best of our knowledge, no previous study has comprehensively investigated the effects of LED light spectra on fruit yield and nutritional quality, including mineral composition, total phenolic content, and antioxidant activity, while simultaneously comparing these responses with those obtained under greenhouse and open-field conditions.

In this context, the objectives of the present study were, first, to evaluate the effects of different LED light spectra (red, blue, and their combination) on the flowering time of okra under a short photoperiod. Second, we assessed fruit characteristics and yield performance. Finally, we investigated the effects of these light spectra on mineral accumulation in the fruits, total phenolic content, and antioxidant activity, and compared these responses with those obtained under natural greenhouse and open-field growing conditions.

## 2 Materials and methods

### 2.1 Plant material and soil properties

Commercial seeds of the okra (*A. esculentus* L.) cultivar Clemson Spineless were obtained from Xotica Collection (Europe). This cultivar was selected because of its stable physiological performance and its sensitivity to environmental conditions, particularly light. It has been widely used in studies investigating the effects of light spectrum, irradiance, and photoperiod on okra growth, development, and productivity (Dada and Adejumo, 2015; Hamamoto and Yamazaki, 2009; Rrrhichaii et al., 2004; Seydou et al., 2023). In addition, Clemson Spineless is an early-maturing cultivar, reaching harvest maturity within approximately 52 and 64 days under spring and summer growing conditions, respectively. Plants were grown in a substrate prepared by mixing 40% composted pig manure with 60% soil (v/v). The physicochemical properties of the substrate were determined prior to the experiment. Soil pH was measured in a soil-water suspension (2:5, w/v) using a pH meter. Total nitrogen was determined using the Kjeldahl method (Kjeldahl, 1883), whereas organic carbon content was quantified following the Walkley and Black procedure (Walkley and Black, 1934). Cation exchange capacity (CEC) was determined using the ammonium acetate extraction method (Thomas, 1982). The concentrations of phosphorus, calcium, magnesium, sodium, and potassium were quantified by inductively coupled plasma optical emission spectrometry (ICP-OES). The main physicochemical characteristics of the substrate, including pH, total nitrogen, organic carbon, phosphorus, calcium, magnesium, sodium, potassium, and CEC, are presented in **Table 1**.

**Table 1:** Physicochemical properties of the growing substrate used in the experiment.

Parameter	Value
pH	6.3
Nitrogen (%)	0.31
Organic carbon (%)	3.7
Phosphorus (mg·kg^-1^)	30.33
Calcium (mg·kg^-1^)	94.29
Magnesium (mg·kg^-1^)	31.34
Sodium (mg·kg^-1^)	63.35
Potassium (mg·kg^-1^)	50.89
CEC (cmol·kg^-1^)	13.4

Soil moisture was monitored throughout the experiment, and irrigation was applied manually whenever the substrate reached the same predetermined moisture threshold across all growing environments. Each pot received 375 mL of water per irrigation event. Under these conditions, irrigation was required approximately every four days for plants grown under LED lighting and greenhouse conditions, whereas outdoor plants required irrigation approximately every two days. Thus, irrigation frequency was not imposed as an independent treatment factor but resulted from differences in water loss among the growing environments.

### 2.2 Experimental design and lighting treatments

The experiment was conducted at the Félix Houphouët-Boigny National Polytechnic Institute, Yamoussoukro, Côte d’Ivoire (6°52.9650′ N, 5°13.7340′ W; 250 m above sea level), from November 8, 2025, to January 7, 2026. Ten okra seeds were sown per pot at a depth of 1 to 3 cm. For the greenhouse (GH) control light treatment, four pots were placed in a culture box covered with transparent plastic. Four pots were exposed directly to sunlight, considered as an outdoor (OD) control. After germination, at 19 days after sowing (DAS), the plants were thinned, retaining only the strongest seedlings per pot. The LED treatments were implemented in three physically separated growing boxes located in the same room, with each box covered with black opaque plastic sheeting and assigned to one spectral treatment (red, blue, or red/blue). Each box contained four okra plants, and each plant was illuminated by an individual LED module corresponding to the assigned spectrum, with the same module-to-plant distance maintained throughout the experiment. The four plants receiving the same spectrum were maintained within the same box to provide a common growing environment. Thus, four plants were evaluated within each treatment-specific growing box. The plants were maintained at fixed positions throughout the experiment and were not rotated or repositioned. These three LED lighting treatments consist of two monochromatic light treatments, blue (B, peak wavelength: 452 nm) and red (R, peak wavelength: 659 nm), whose lighting system was previously described by Yao et al (Yao et al., 2026), and one combined red-blue treatment (RB 9:1) consisting of red (657 nm) and blue (447 nm) wavelengths at a 9:1 ratio. The selection of the 9:1 red-to-blue ratio was guided by both previous literature and our previous findings in okra. In our previous study, red light alone accelerated flowering in okra, a quantitative short-day plant, compared with blue light and alternating red/blue treatments (Yao et al., 2025). Given the short 10-h photoperiod used in the present study, we therefore selected a red-dominant spectrum containing 90% red light and a small proportion of blue light to investigate whether this combination could promote early flowering while retaining the potential complementary effects of blue light. Previous studies have also investigated the R90B10 spectrum. In *Hippeastrum hybridum*, R90B10 promoted biomass accumulation in bulbs, leaves, and flowers, although it delayed flowering compared with R10B90 and the control (Wang et al., 2022). In cut roses, R90B10 improved growth, photosynthetic performance, and yield and accelerated flowering compared with the control treatment (Davarzani et al., 2023). Thus, the RB 9:1 treatment was selected as a red-dominant spectrum combining the well-established roles of red and blue light, rather than as part of a systematic optimization of different red-to-blue ratios. The normalized spectra of LED modules are shown in **Figure 1**.

**Figure 1:**
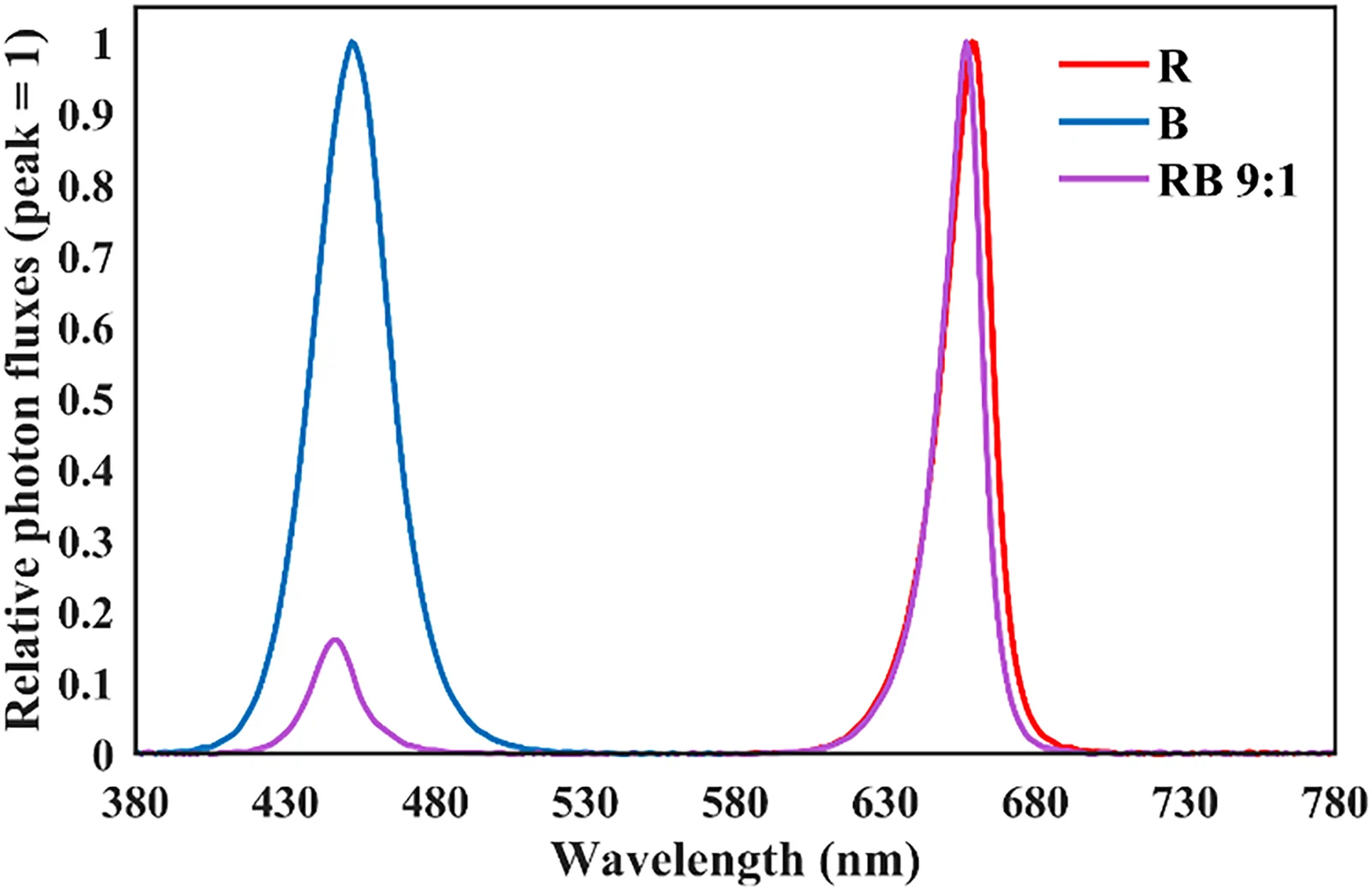
Spectral distribution of the blue (B), red (R), and red–blue (RB 9:1) LED modules used for the cultivation of *A. esculentus* under LED lighting.

All LED treatments were applied under a 10-h photoperiod with a target photosynthetic photon flux density (PPFD) of 400 µmol·m^−2^·s^−1^. This photoperiod was selected based on previous studies demonstrating that short-day conditions promote floral induction, anthesis, and fruit production in okra (Maduagwuna et al., 2019; Nwoke, 1983). Moreover, photoperiods ranging from 10 to 12.28 h have been reported to maximize pod yield, whereas longer photoperiods (14–16 h) markedly reduce productivity (Maduagwuna et al., 2019). Therefore, a 10-h photoperiod was adopted to promote flowering while minimizing the energy consumption associated with artificial lighting. The PPFD at the canopy level was measured using a LightScout 3415FXSE quantum sensor. To ensure comparable irradiance among LED treatments, the distance between the LED fixtures and the plant canopy was adjusted regularly throughout the experiment. Under these conditions, the daily light integral (DLI) was maintained at 14.4 mol·m^−2^·d^−1^ for all LED treatments.

### 2.3 Outdoor and greenhouse light conditions

Outdoor and greenhouse light conditions were continuously monitored using a custom Internet of Things (IoT)-based acquisition system consisting of an ESP32 microcontroller coupled with two digital light sensors (SEN0390, DFRobot, Shanghai, China). Illuminance was automatically recorded every 5 min from 06:00 to 18:30, covering the daylight period throughout the experiment and stored in a local database for subsequent processing. Since illuminance (lux) does not directly represent photosynthetically active radiation, all measurements were converted to photosynthetic photon flux density (PPFD, µmol·m^−2^·s^−1^). A conversion coefficient was established by simultaneously recording illuminance with the SEN0390 sensors and PPFD with a calibrated LightScout quantum sensor (Spectrum Technologies Inc., Aurora, IL, USA) operating in solar mode. Measurements were performed at different periods of the day to account for changes in natural irradiance. The average conversion coefficient obtained was 0.0168, which was subsequently applied to all recorded illuminance values. This coefficient was close to the value commonly reported in the literature (0.0185) (Apogee Instruments, n.d.), with the slight difference likely resulting from sensor spectral characteristics and the prevailing environmental conditions.

The average daily variation in PPFD during the experimental period is presented in **Figure 2**. Over the entire cultivation period, the mean PPFD was 343.25 ± 54.33 µmol·m^−2^·s^−1^ under outdoor conditions and 233.25 ± 35.61 µmol·m^−2^·s^−1^ under greenhouse conditions. The minimum and maximum PPFD values recorded under outdoor conditions were 211.91 and 486.83 µmol·m^−2^·s^−1^, respectively, whereas those under greenhouse conditions were 145.81 and 305.94 µmol·m^−2^·s^−1^, respectively (**Table 2**). **Figure 3** presents the variation in daily photoperiod throughout the experimental period, calculated from daily sunrise and sunset times for Yamoussoukro obtained from an online astronomical database (Sunrise and Sunset in Yamoussoukro, Ivory Coast, 2026). The mean photoperiod during the cultivation period was 11.43 ± 0.02 h. By combining the recorded photoperiod with the corresponding PPFD values, the daily light integral (DLI) was calculated, and its temporal variation is shown in **Figure 4**. Over the entire experimental period, the mean DLI was 14.13 ± 2.24 mol·m^−2^·d^−1^ under outdoor conditions and 9.60 ± 1.47 mol·m^−2^·d^−1^ under greenhouse conditions. Values are expressed as mean ± standard deviation.

**Figure 2:**
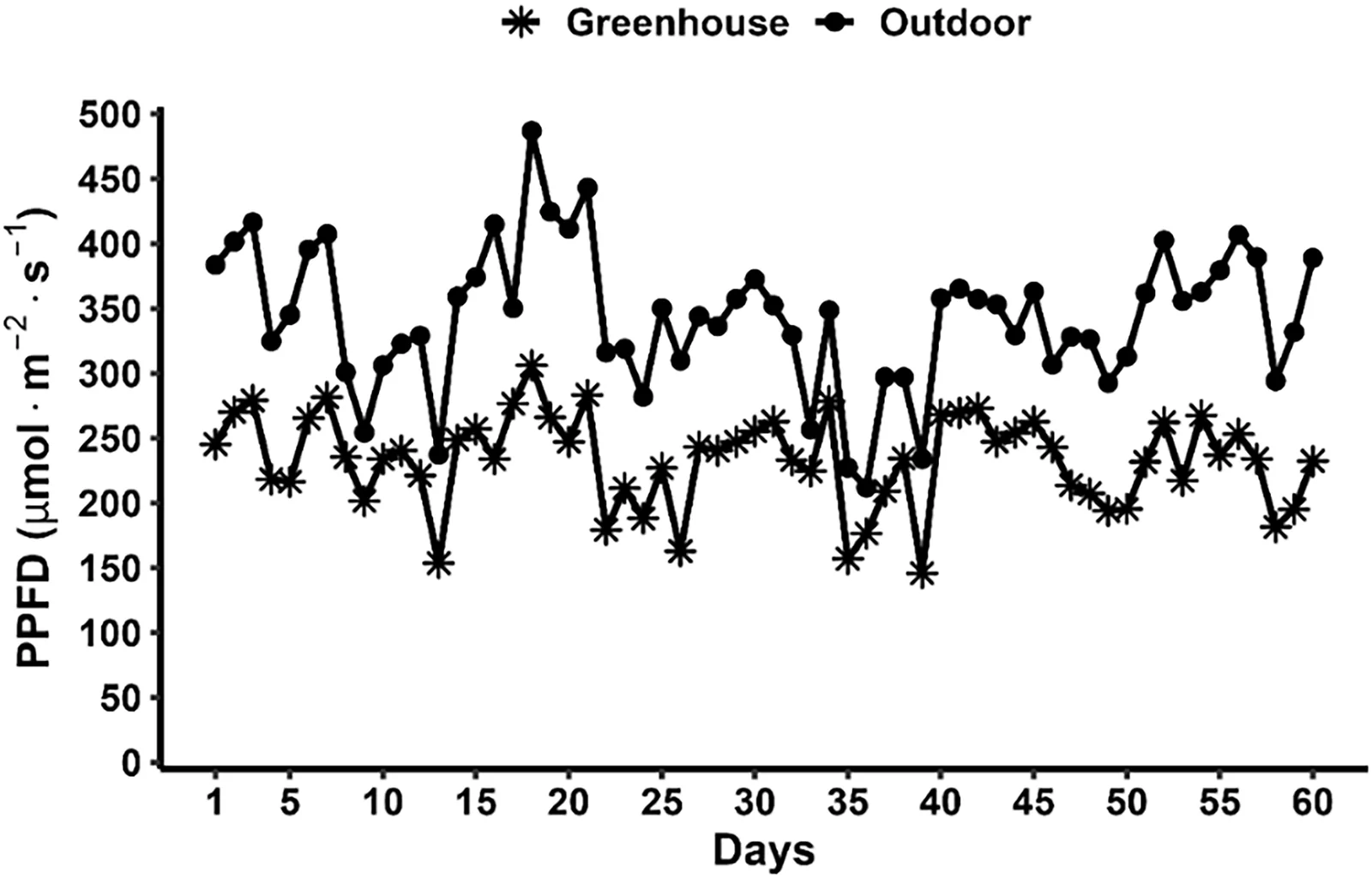
Average daily photosynthetic photon flux density (PPFD) under outdoor and greenhouse conditions during the experimental period.

**Table 2:** Minimum, mean ± standard deviation (SD), and maximum photosynthetic photon flux density (PPFD) under greenhouse and outdoor conditions during the 60-day experimental period.

Growing environment	Minimum (µmol·m^−2^·s^−1^)	Mean ± SD (µmol·m^−2^·s^−1^)	Maximum (µmol·m^−2^·s^−1^)
Outdoor	211.91	343.25 ± 54.33	486.83
Greenhouse	145.81	233.25 ± 35.61	305.94

**Figure 3:**
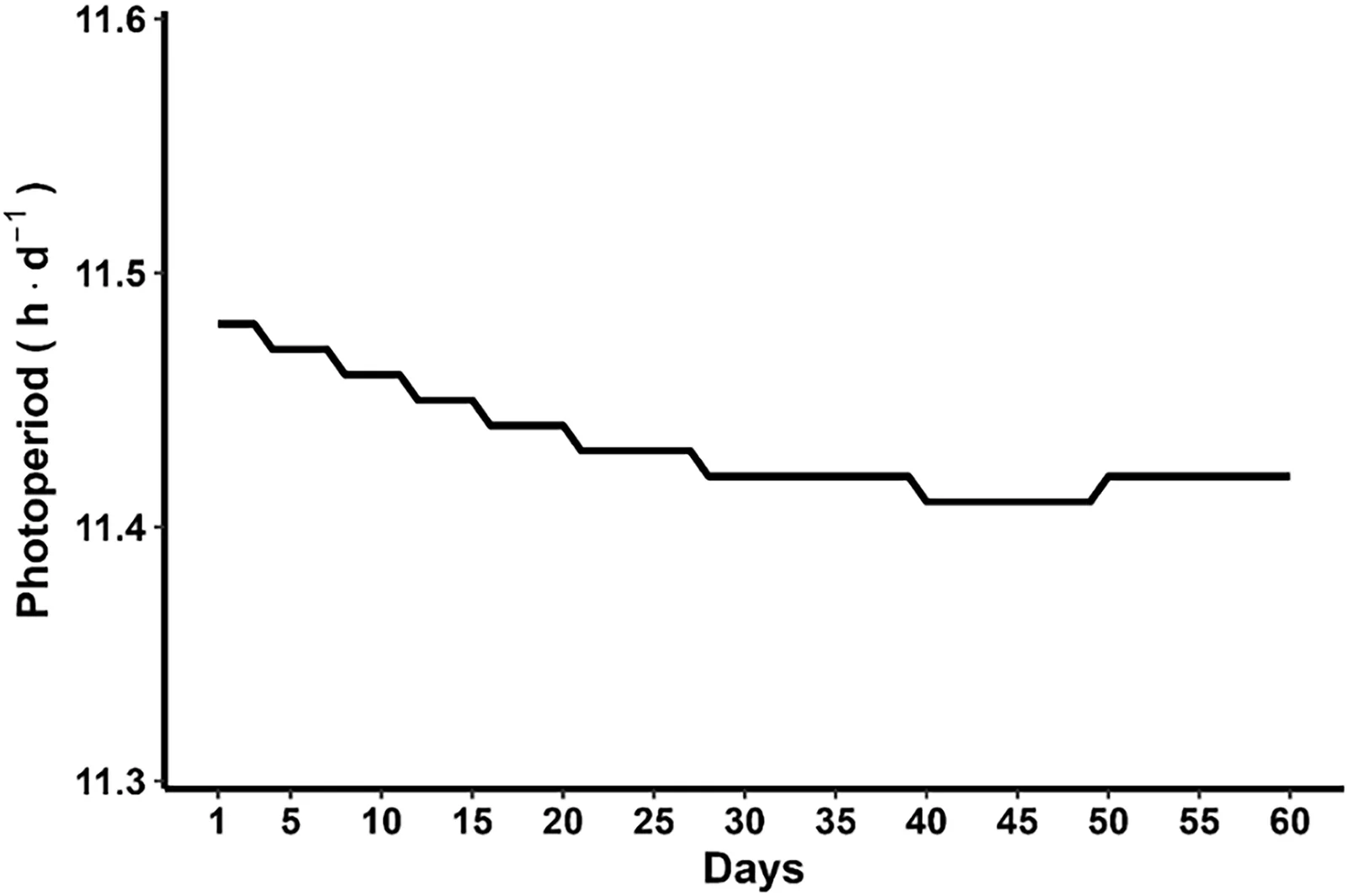
Daily variation in photoperiod during the experimental period in Yamoussoukro, Côte d’Ivoire.

**Figure 4:**
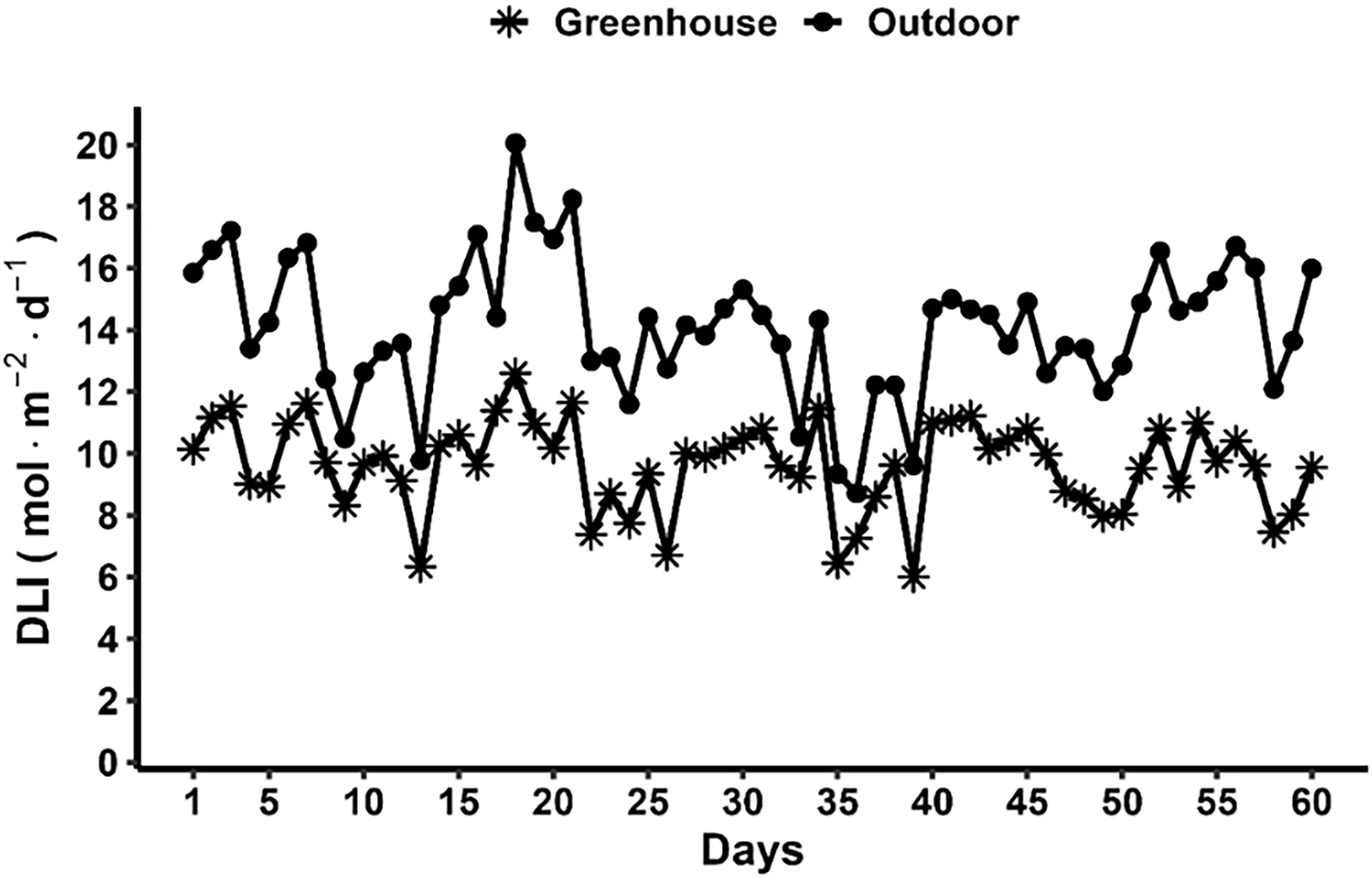
Daily light integral (DLI) under outdoor and greenhouse conditions throughout the experimental period.

### 2.4 Temperature and relative humidity under different growing conditions

Air temperature and relative humidity were continuously monitored using the IoT-based environmental monitoring system previously described by Yao et al (Yao et al., 2025). Temperature and relative humidity were recorded throughout the 60-day cultivation period and analyzed over successive 24-h cycles. The daily variations in air temperature and relative humidity are presented in **Figures 5**, **6**, respectively, while the corresponding mean values for each growing condition over the entire cultivation period are presented as mean ± standard deviation (Mean ± SD). Air temperature remained relatively stable across all lighting treatments, ranging from 28.27 ± 0.85 °C in the greenhouse to 29.04 ± 0.64 °C under B light. Mean temperatures under R (28.73 ± 0.65 °C) and RB 9:1 (28.54 ± 0.62 °C) LED lighting were comparable to those measured under outdoor (28.45 ± 1.00 °C) and greenhouse conditions. In contrast, relative humidity showed clear differences between the controlled LED environments and the natural growing conditions. Mean relative humidity values under B, R, and RB9:1 LED lighting were 46.63 ± 2.30%, 46.65 ± 2.21%, and 47.88 ± 2.29%, respectively, whereas considerably higher values were recorded in the greenhouse (74.40 ± 2.81%) and under outdoor conditions (72.27 ± 3.85%).

**Figure 5:**
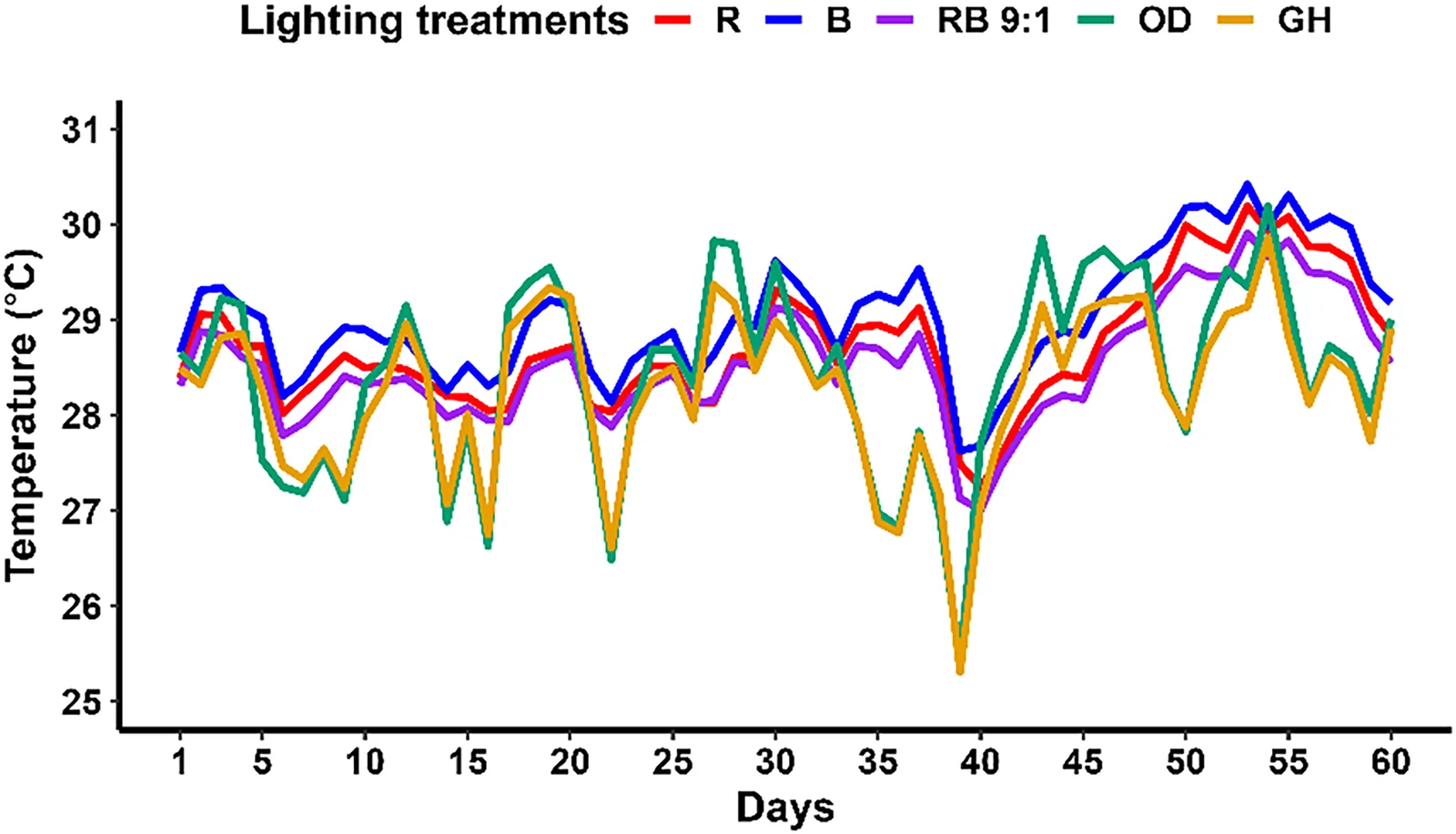
Daily variation in air temperature under red (R), blue (B), red–blue (RB 9:1), outdoor (OD), and greenhouse (GH) conditions.

**Figure 6:**
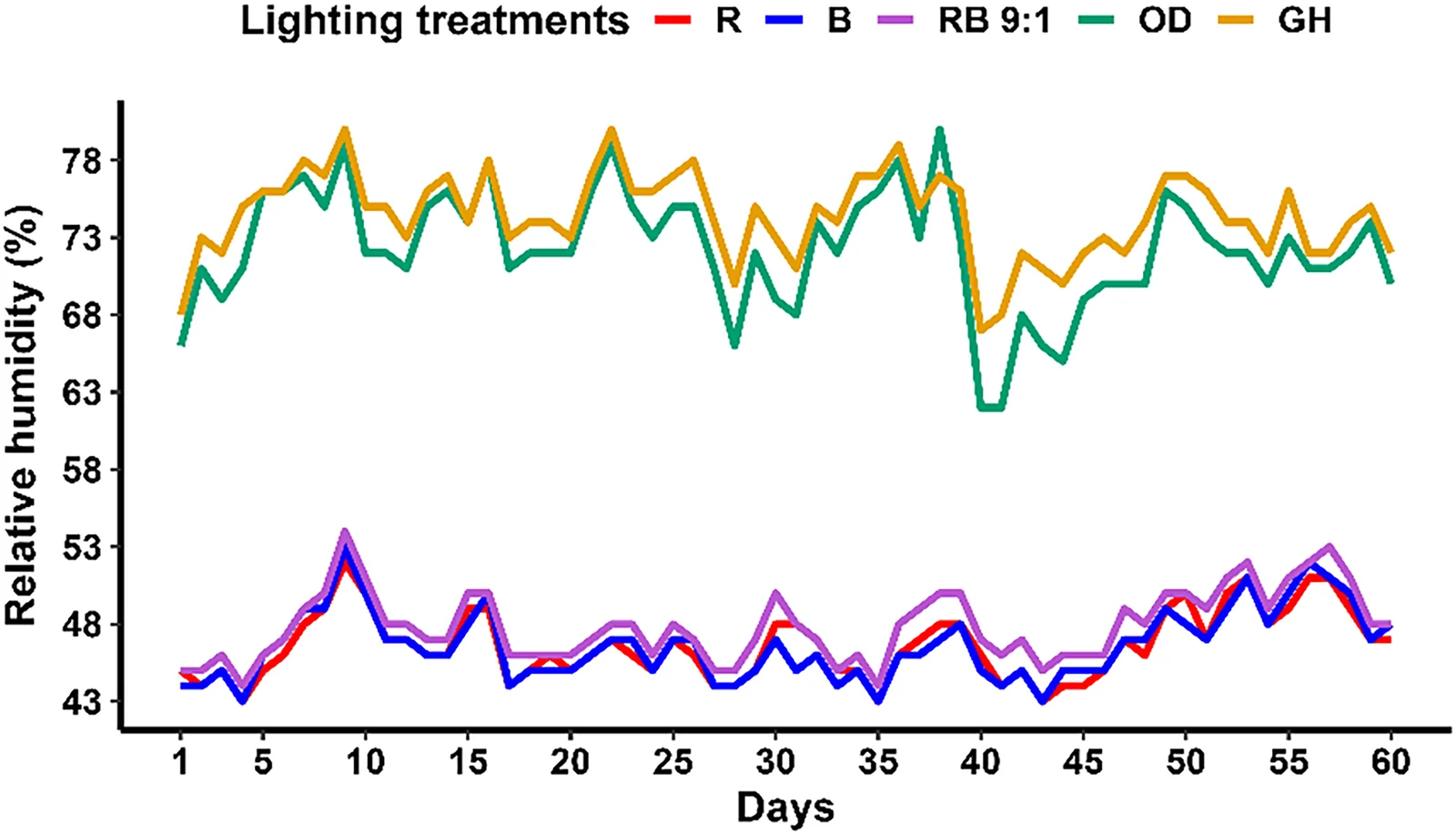
Daily variation in relative humidity under red (R), blue (B), red–blue (RB 9:1), outdoor (OD), and greenhouse (GH) conditions.

### 2.5 Flowering characteristics

Parameters related to floral bud initiation and flowering were assessed by visual observation. The date of the first floral bud appearance, the date of the first flower opening, and the node bearing the first floral bud were recorded for each plant.

### 2.6 Fruit morphology and yield assessment

Fruits were harvested every six days from the onset of flowering to prevent excessive fruit maturation. At the end of the 60-day experimental period, no new floral buds were observed on plants under any of the lighting treatments. Fruits were harvested at the immature edible stage, which corresponds to the stage at which okra fruits are commonly consumed. All fruits harvested from each individual plant during the experimental period were pooled within plant, dried, and ground together to obtain one homogeneous powder per plant. The resulting powder from each plant was divided into three portions for subsequent DPPH, TPC, and XRF analyses. Thus, four biological samples were obtained for each treatment, corresponding to the four individual plants. The fresh mass of all harvested fruits was recorded and included in the calculation of fruit yield. The first three fruits harvested from each plant were used for the evaluation of morphological quality parameters. The measured parameters included fruit length, fruit diameter, fresh weight, and dry weight. Fruit diameter was measured at the midpoint of the fruit using a digital caliper, while fruit length was measured with a graduated ruler from the junction between the fruit and the base of the peduncle. Fresh and dry fruit weights (FW and DW) were measured using a precision balance (± 0.01 g). Dry weight was determined after oven-drying the fresh fruits at 70 °C for 48 h. From the fourth fruit onward, fruits were only counted and weighed. The total number of fruits per plant was used to determine the yield per plant, whereas the yield per hectare was estimated from the total fruit mass produced per plant multiplied by the planting density. The yield per plant was calculated using Equation 1:

(1) $$\text{Y}\text{i}\text{e}\text{l}\text{d}\;\text{p}\text{e}\text{r}\;\text{p}\text{l}\text{a}\text{n}\text{t}\left(\text{g}\cdot \text{p}\text{l}\text{a}\text{n}{\text{t}}^{-1}\right)=\text{N}\text{u}\text{m}\text{b}\text{e}\text{r}\;\text{o}\text{f}\;\text{f}\text{r}\text{u}\text{i}\text{t}\text{s}\times \text{M}\text{e}\text{a}\text{n}\;\text{f}\text{r}\text{u}\text{i}\text{t}\;\text{m}\text{a}\text{s}\text{s}\left(\text{g}\right)$$

Estimated yield per hectare was estimated from the total fruit mass produced per plant multiplied by the planting density and expressed in tons per hectare (Equation 2). Four plants were grown on a total area of 0.36 m^2^, corresponding to a planting area of 0.09 m^2^ per plant (0.3 m × 0.3 m).

(2) $$\text{E}\text{s}\text{t}\text{i}\text{m}\text{a}\text{t}\text{e}\text{d}\;\text{y}\text{i}\text{e}\text{l}\text{d}\;\text{p}\text{e}\text{r}\;\text{h}\text{e}\text{c}\text{t}\text{a}\text{r}\text{e}\left(\text{t}\cdot \text{h}{\text{a}}^{-1}\right)=\frac{{\text{M}}_{\text{p}\text{l}\text{a}\text{n}\text{t}}\left(\text{k}\text{g}\right)\times \text{D}\left(\text{p}\text{l}\text{a}\text{n}\text{t}\text{s}\cdot \text{h}{\text{a}}^{-1}\right)}{1000}$$

Where *M_plant_* is the fruit mass per plant (kg) and *D* is the planting density (plants·ha^−1^).

Fruit water content was calculated using Equation 3:

(3) $$\text{F}\text{r}\text{u}\text{i}\text{t}\;\text{w}\text{a}\text{t}\text{e}\text{r}\;\text{c}\text{o}\text{n}\text{t}\text{e}\text{n}\text{t}\left(\%\right)=\frac{\text{F}\text{W}-\text{D}\text{W}}{\text{F}\text{W}}\times 100$$

### 2.7 Antioxidant activity of okra fruits (DPPH assay)

The antioxidant activity of immature okra (*A. esculentus* L.) fruits was evaluated using the 2,2-diphenyl-1-picrylhydrazyl (DPPH) radical scavenging assay. DPPH is a stable free radical commonly used to assess the free radical scavenging capacity of antioxidant compounds by measuring the decrease in absorbance at 517 nm. Fruits were washed and oven-dried at 70 °C for 48 h before being finely ground and sieved through a 180 μm mesh. The resulting powder was extracted with 80% methanol by mixing 1 g of powder with 10 mL of solvent. The mixture was stirred at room temperature for 24 h and then centrifuged at 5,000 rpm for 10 min. The clear supernatant was collected and used as the plant extract for subsequent analyses. The DPPH assay was performed according to a previously described method (Velazquez et al., 2003), with slight modifications. Briefly, a 0.1 mM DPPH solution was prepared by dissolving 3.94 mg of DPPH in 100 mL of methanol. Subsequently, 1.5 mL of the DPPH solution was mixed with 0.75 mL of the plant extract, vortexed thoroughly, and incubated in the dark at room temperature for 15 min to obtain the sample solution (AS). Absorbance was then measured at 517 nm using a Jenway Model 741001 spectrophotometer (Cole-Parmer Ltd, Stone, Staffs, UK). The blank (AB) consisted of a mixture containing 1.5 mL of DPPH solution and 0.75 mL of methanol. All measurements were performed in triplicate. Antioxidant activity was expressed as the percentage of DPPH radical inhibition was calculated using Equation 4:

(4) $$\text{D}\text{P}\text{P}\text{H}\;\text{r}\text{a}\text{d}\text{i}\text{c}\text{a}\text{l}\;\text{i}\text{n}\text{h}\text{i}\text{b}\text{i}\text{t}\text{i}\text{o}\text{n}\left(\%\right)=\frac{\left(\text{A}\text{B}-\text{A}\text{S}\right)}{\text{A}\text{B}}\times 100$$

where AS is the absorbance of the sample (DPPH solution + plant extract) and AB is the absorbance of the blank (DPPH solution + methanol).

### 2.8 Total phenolic content

Total phenolic content (TPC) was determined using the Folin–Ciocalteu method with gallic acid as the calibration standard (Singleton et al., 1999). Briefly, 1 g of dried okra fruit powder was mixed with 10 mL of 80% methanol. The mixture was stirred at room temperature for 24 h and then centrifuged at 5,000 rpm for 10 min. The resulting supernatant was collected and used as the plant extract for subsequent analyses. Prior to analysis, the extracts were diluted 50-fold (1:50). For TPC determination, the reaction mixture consisted of 400 μL of diluted extract, 2.0 mL of Folin–Ciocalteu reagent, and 1.6 mL of 7% sodium carbonate (Na_2_CO_3_) solution. After vortex mixing, the reaction mixture was incubated in the dark at room temperature for 2 h, and the absorbance was measured at 765 nm using a Jenway Model 741001 spectrophotometer (Cole-Parmer Ltd, Stone, Staffs, UK). Quantification was performed using a calibration curve prepared with gallic acid standard solutions (10–100 mg L^−1^) in 80% methanol. All analyses were performed in triplicate, and the results were expressed as milligrams of gallic acid equivalents per gram of dry matter (mg GAE g^−1^ DW). Total phenolic content was calculated using Equation 5:

(5) $$\text{T}\text{P}\text{C}=\frac{\text{C}\times \text{V}\times \text{D}\text{F}}{\text{m}}$$

where C is the concentration obtained from the calibration curve (mg L^−1^), V is the total extraction volume (0.01 L), DF is the dilution factor (50), and m is the dry mass of the sample (1 g).

### 2.9 Determination of mineral composition by X-ray fluorescence spectrometry

Elemental composition was determined using a portable X-ray fluorescence (XRF) spectrometer (Niton XL3t; Thermo Fisher Scientific, USA) equipped with a sample stand for solid materials. Fruits collected from each individual plant were pooled within plant, dried, and ground separately, resulting in four biological samples per treatment. For XRF analysis, 1 g of dried okra powder was placed into XRF sample cups and sealed with 6 μm Mylar film to minimize analytical interference and prevent external contamination. Each biological sample was analyzed individually in both Soil and Mining modes. The measurement time was 90 s in Soil mode and 120 s in Mining mode. Three measurements were performed for each sample, and the repeated measurements were averaged to obtain one value per biological sample. The quantified elements included the macronutrients calcium (Ca), potassium (K), phosphorus (P), and sulfur (S); the micronutrients chlorine (Cl), copper (Cu), iron (Fe), molybdenum (Mo), and zinc (Zn); and the trace elements chromium (Cr), rubidium (Rb), silicon (Si), and strontium (Sr). Elemental concentrations were expressed on a dry-weight basis (mg kg^−1^ DW). The instrument output provided measurement-error estimates. According to the manufacturer, Niton XRF analyzers provide two-sigma precision estimates corresponding to a 95% confidence interval, while instrumental limits of detection (LODs) are reported for specified analytical conditions and reference matrices. Because these manufacturer-reported LODs depend on measurement conditions and matrix composition, they were not considered as matrix-specific LODs for dried okra fruit. Concentrations below the instrumental detection limit were therefore reported as “< LOD”. No plant-matrix-specific calibration, certified reference material for the okra fruit matrix, recovery experiment, or experimental limit of quantification (LOQ) determination was performed. Consequently, the XRF results were interpreted as comparative elemental measurements obtained under the specified analytical conditions rather than as fully validated quantitative nutritional composition.

### 2.10 Statistical analysis

Four okra plants were evaluated within each growing environment (*n = 4*), with the four plants sharing the same treatment-specific growing unit. For biochemical and elemental analyses, each plant provided one biological sample, and technical replicate measurements were averaged and were not treated as independent biological replicates.

Statistical analyses were performed using R software (version 4.4.2; RStudio, Boston, MA, USA). The normality of model residuals was assessed using the Shapiro–Wilk test, and homogeneity of variances was evaluated using Levene’s test. Given the small number of biological replicates, the results of these diagnostic tests were interpreted cautiously. When the assumptions for parametric analysis were considered adequately satisfied, a one-way analysis of variance (ANOVA) was performed, followed by Tukey’s honestly significant difference (HSD) *post hoc* test. When these assumptions were not adequately satisfied, the non-parametric Kruskal–Wallis test was applied, followed by Dunn’s *post hoc* test with Bonferroni correction for multiple comparisons. Differences were considered statistically significant at *p< 0.05*.

## 3 Results

### 3.1 Effects of light treatments on flowering characteristics

Statistical analysis revealed a highly significant effect of light treatments on the day of first floral bud initiation (**Figure 7A**). Plants grown under the RB 9:1 treatment initiated floral buds significantly earlier (23.00 ± 0.41 DAS) than those grown under greenhouse condition (31.50 ± 0.87 DAS) or under B treatment (28.25 ± 0.85 DAS). In contrast, the R (26.00 ± 0.58 DAS) and outdoor (26.50 ± 1.19 DAS) treatments exhibited intermediate values and did not differ significantly from either the RB 9:1 or B treatments.

**Figure 7:**
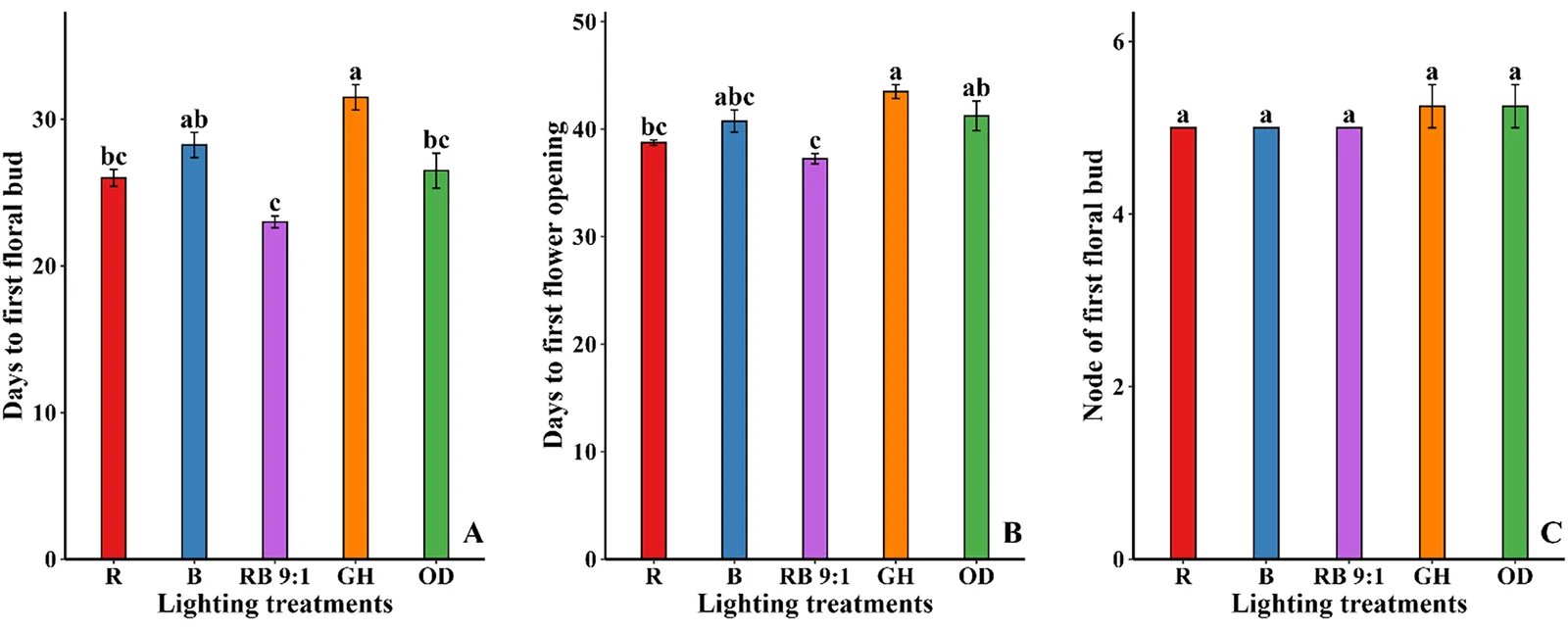
Flowering characteristics of okra (*A. esculentus*) plants grown under red (R), blue (B), and red-blue (RB 9:1) LED light, greenhouse (GH), and outdoor (OD) conditions. **(A)** Days to first floral bud initiation, **(B)** days to first flower opening, and **(C)** node bearing the first floral bud. Bars represent mean values ± standard error (SE) (*n = 4*). Different letters indicate significant differences among treatments according to Tukey’s HSD test or Dunn’s *post hoc* test (*p< 0.05*).

Light treatments also significantly affected the day of first flower opening (**Figure 7B**). The RB 9:1 treatment promoted the earliest flower opening (37.25 ± 0.48 DAS), significantly earlier than the outdoor (41.25 ± 1.38 DAS) and greenhouse (43.50 ± 0.65 DAS) treatments. Similarly, plants grown under the R treatment reached first flower opening earlier (38.75 ± 0.25 DAS) than those grown under greenhouse condition, whereas the B treatment (40.75 ± 1.03 DAS) showed intermediate values and did not differ significantly from the other treatments.

In contrast, the node bearing the first floral bud was not significantly affected by light treatments (**Figure 7C**), with floral buds consistently initiated at the fifth or sixth node across all treatments. Representative images illustrating floral bud initiation and flower development of okra plants under the different light treatments are presented in **Figure 8**.

**Figure 8:**
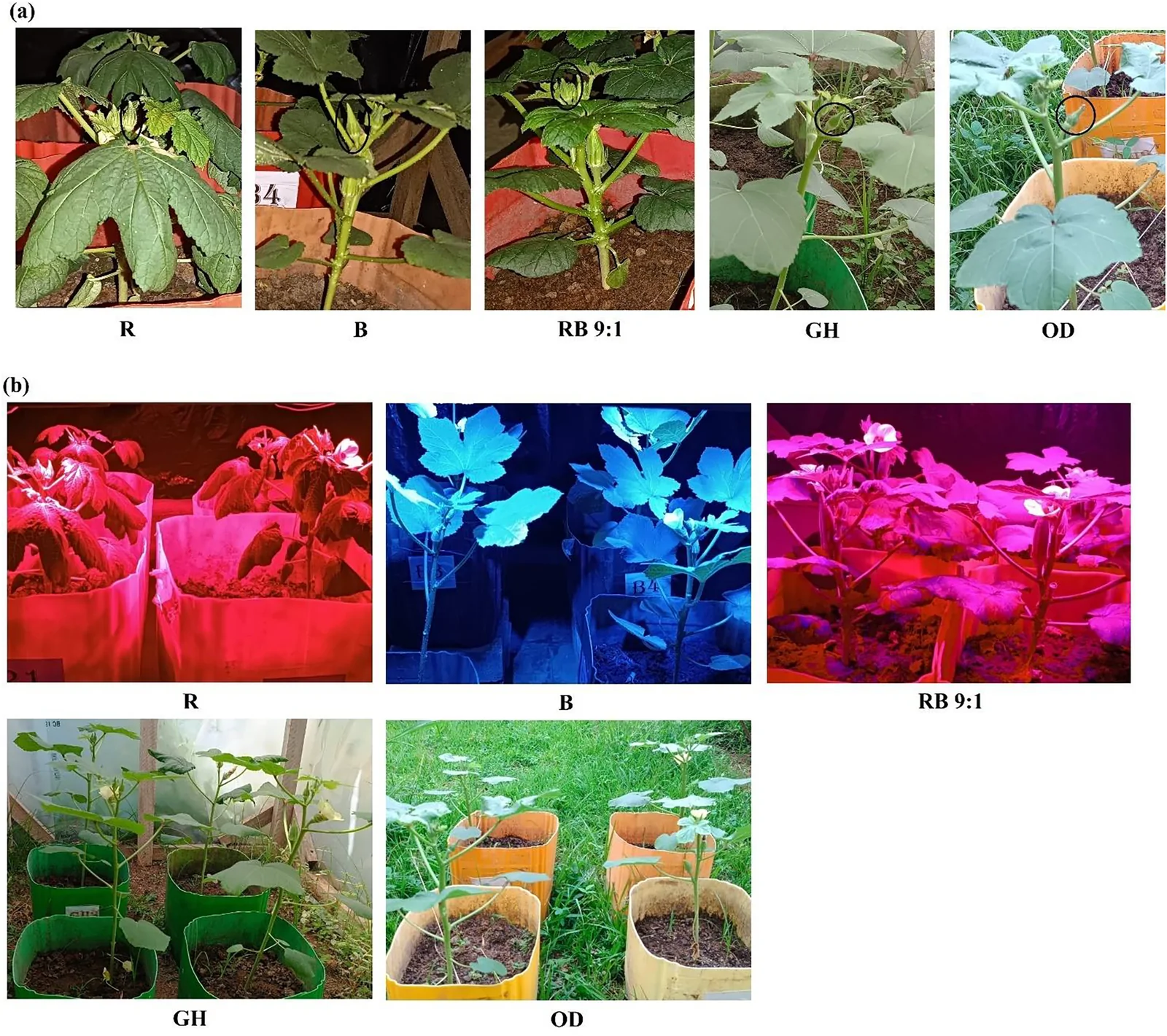
Representative images of floral bud development and flowering in okra (*A. esculentus* cv. Clemson Spineless) plants grown under red (R), blue (B), and red-blue (RB 9:1) LED light, greenhouse (GH), and outdoor (OD) conditions. **(a)** Close-up images of floral buds from representative plants at 41 days after sowing (DAS), with the LED lighting switched off to facilitate their visualization. **(b)** Representative images of flowering plants under the five growing conditions.

### 3.2 Effects of light treatments on fruit morphology and yield

Growing environment had a highly significant effect on fruit length (**Figures 9A**, **10**). Fruit length was greatest under the RB 9:1 treatment (7.12 ± 0.20 cm), which produced significantly longer fruits than the R (6.18 ± 0.14 cm) and B (5.46 ± 0.12 cm) treatments. Fruit length under greenhouse (6.76 ± 0.27 cm) and outdoor (6.39 ± 0.12 cm) conditions did not differ significantly from that under RB 9:1. The B treatment showed the lowest numerical fruit length (5.46 ± 0.12 cm), although it did not differ significantly from the R treatment.

**Figure 9:**
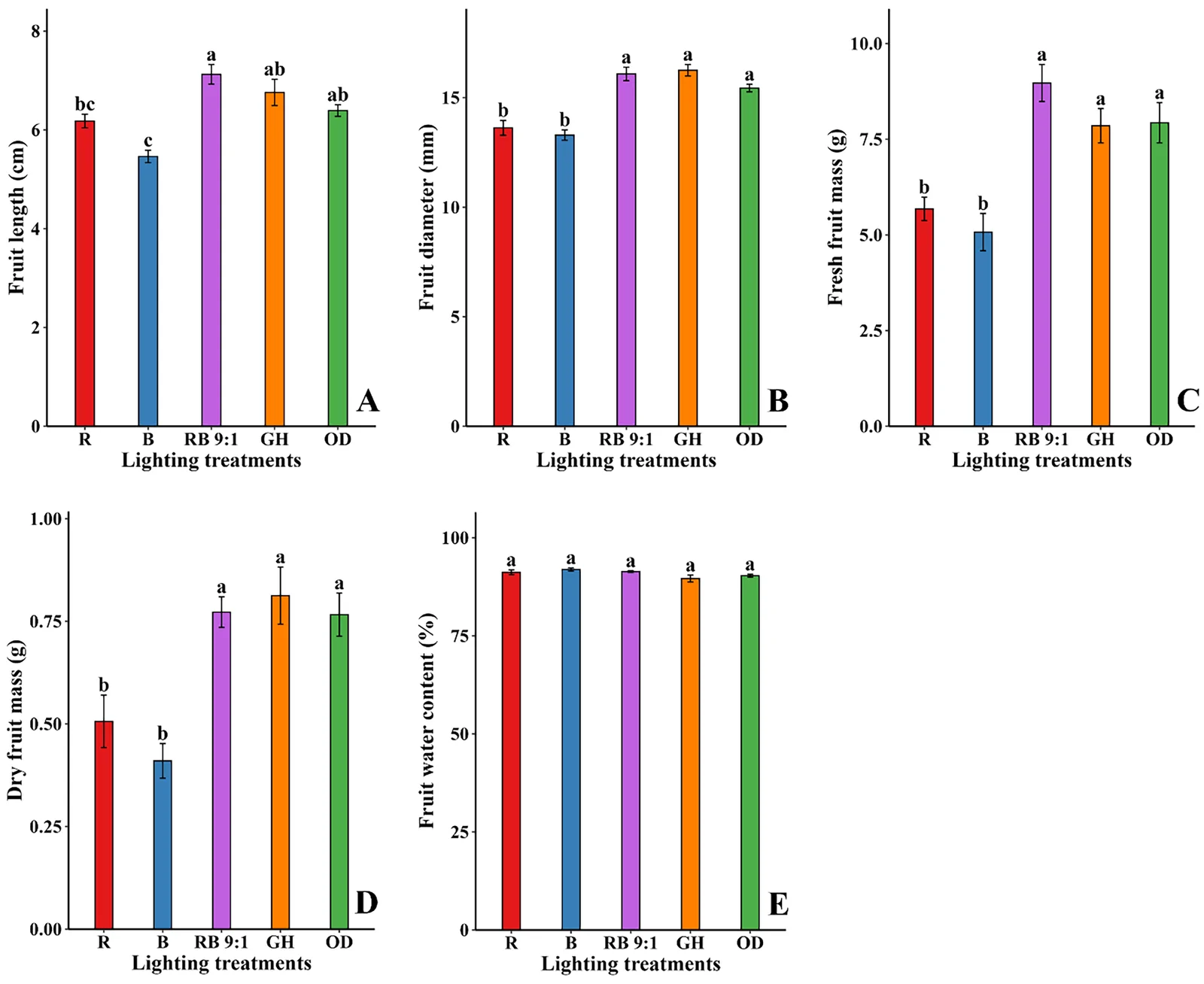
Fruit morphological characteristics of okra (*A. esculentus* L.) plants grown under red (R), blue (B), and red-blue (RB 9:1) LED light, greenhouse (GH), and outdoor (OD) conditions. **(A)** Fruit length, **(B)** fruit diameter, **(C)** fresh fruit mass, **(D)** dry fruit mass, and **(E)** fruit water content. Bars represent mean values ± standard error (SE) (*n = 4*). Different letters indicate significant differences among treatments according to Tukey’s HSD test (*p< 0.05*).

**Figure 10:**
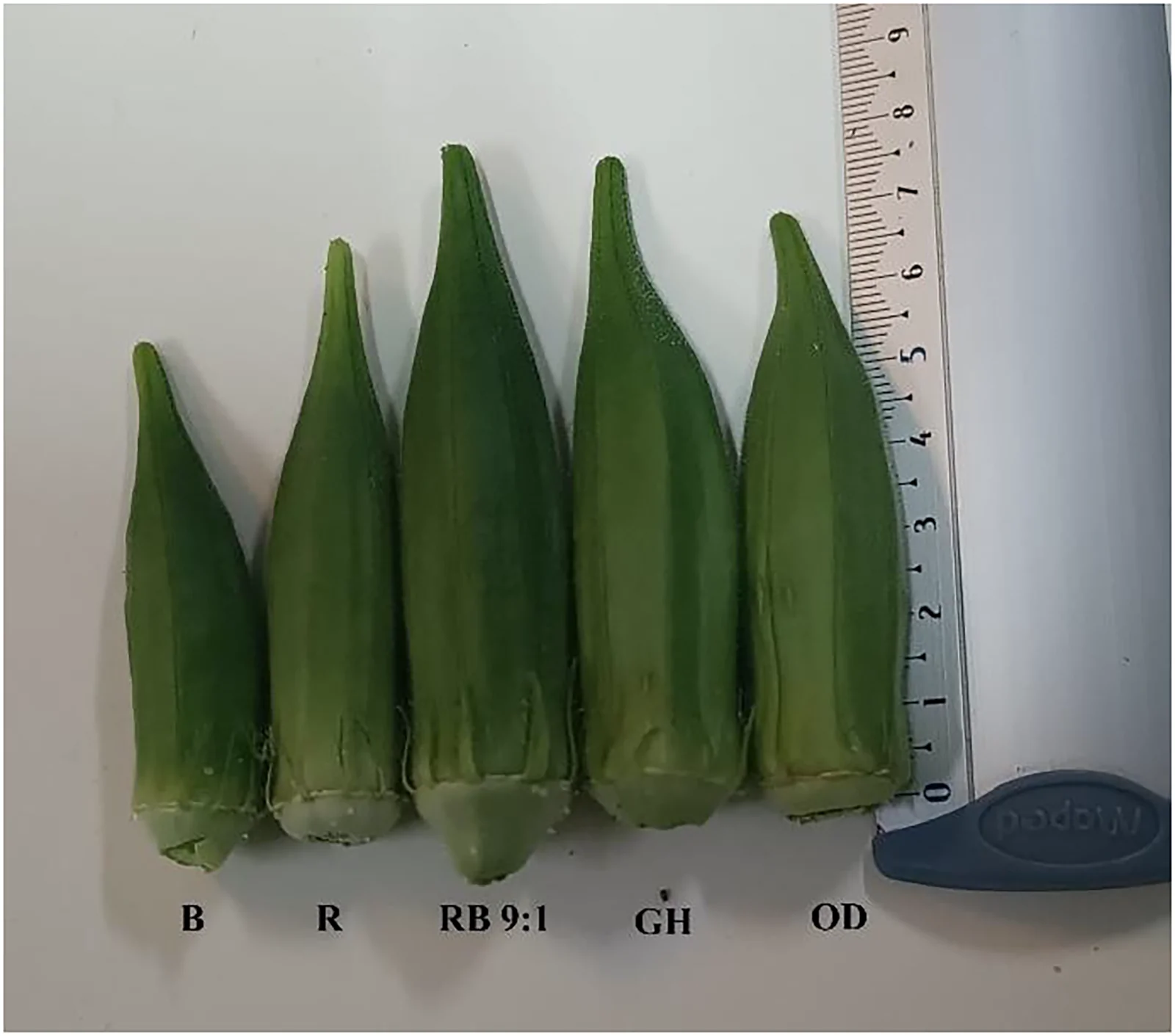
Representative images of okra (*A. esculentus* cv Clemson Spineless) fruits harvested six days after flower opening under different light treatments: red (R), blue (B), and red-blue (RB 9:1) LED light, greenhouse (GH), and outdoor (OD) conditions.

Similarly, fresh fruit mass was significantly affected by the growing environment (**Figure 9C**). RB 9:1 showed the highest numerical mean fresh fruit mass (8.97 ± 0.48 g fruit^−1^), although it did not differ significantly from the outdoor (7.93 ± 0.53 g fruit^−1^) and greenhouse (7.85 ± 0.45 g fruit^−1^) treatments. Fresh fruit mass under these three treatments was significantly higher than under the R (5.68 ± 0.31 g fruit^−1^) and B (5.07 ± 0.49 g fruit^−1^) treatments, which did not differ significantly from each other.

Fruit diameter (**Figures 9B**, **10**) and dry fruit mass (**Figure 9D**) showed a similar statistical pattern. Greenhouse plants had the highest numerical mean fruit diameter (16.25 ± 0.26 mm) and dry fruit mass (0.81 ± 0.07 g fruit^−1^), followed by RB 9:1 (16.08 ± 0.31 mm; 0.77 ± 0.04 g fruit^−1^) and outdoor plants (15.44 ± 0.17 mm; 0.77 ± 0.05 g fruit^−1^). These three environments did not differ significantly from one another for either trait, but all showed significantly greater fruit diameter and dry fruit mass than the R (13.62 ± 0.34 mm; 0.51 ± 0.06 g fruit^−1^) and B (13.29 ± 0.24 mm; 0.41 ± 0.04 g fruit^−1^) treatments, which did not differ significantly from each other.

In contrast, fruit water content was not significantly affected by the light treatments (**Figure 9E**), with values ranging from 89.61 ± 0.88% under greenhouse conditions to 91.91 ± 0.40% under the B treatment. Representative images of fruit development in okra plants grown under the different light treatments are presented in **Figure 11**.

**Figure 11:**
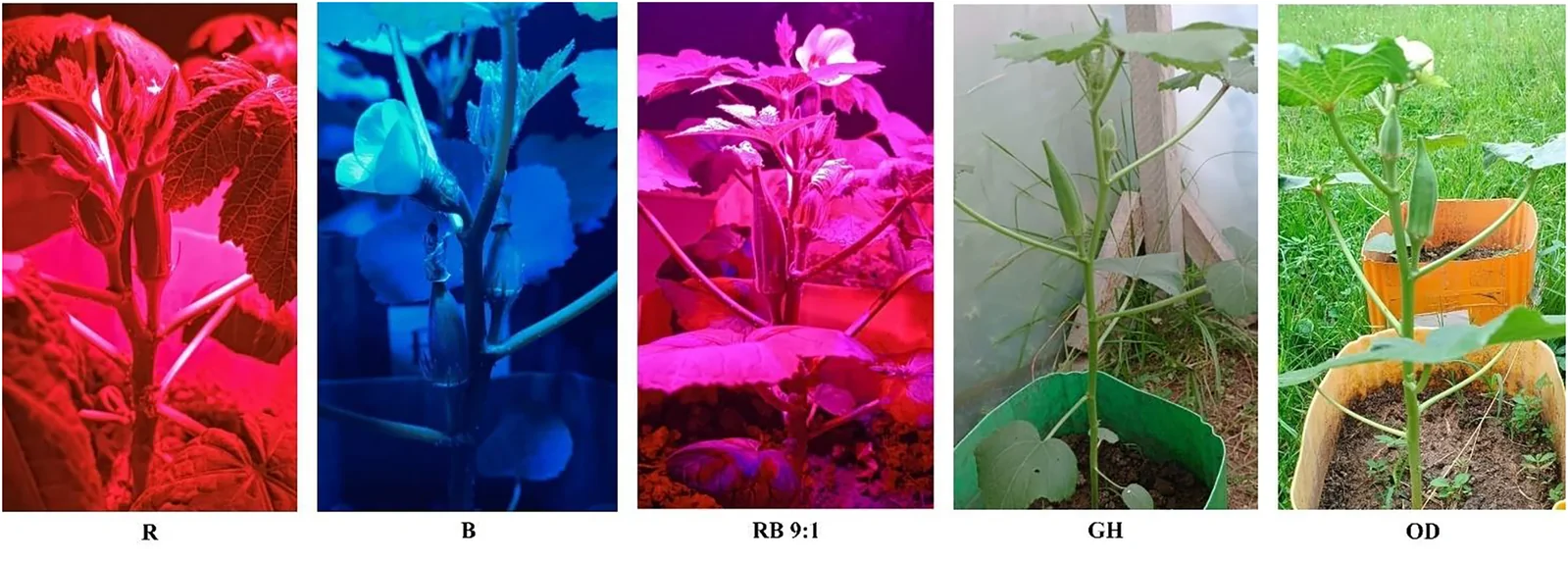
Representative images of fruit development in okra (*A. esculentus* cv. Clemson Spineless) plants grown under red (R), blue (B), and red-blue (RB 9:1) LED light, greenhouse (GH), and outdoor (OD) conditions.

The number of fruits per plant was significantly affected by the light treatments (**Figure 12A**). The RB 9:1 treatment produced the highest number of fruits per plant (5.75 ± 0.25 fruits plant^−1^), which was significantly greater than those obtained under the greenhouse (3.00 ± 0.41 fruits plant^−1^) and outdoor (2.75 ± 0.25 fruits plant^−1^) treatments. In contrast, the R (3.75 ± 0.25 fruits plant^−1^) and B (3.75 ± 0.25 fruits plant^−1^) treatments showed intermediate values and did not differ significantly from either the RB 9:1 or the greenhouse and outdoor treatments.

**Figure 12:**
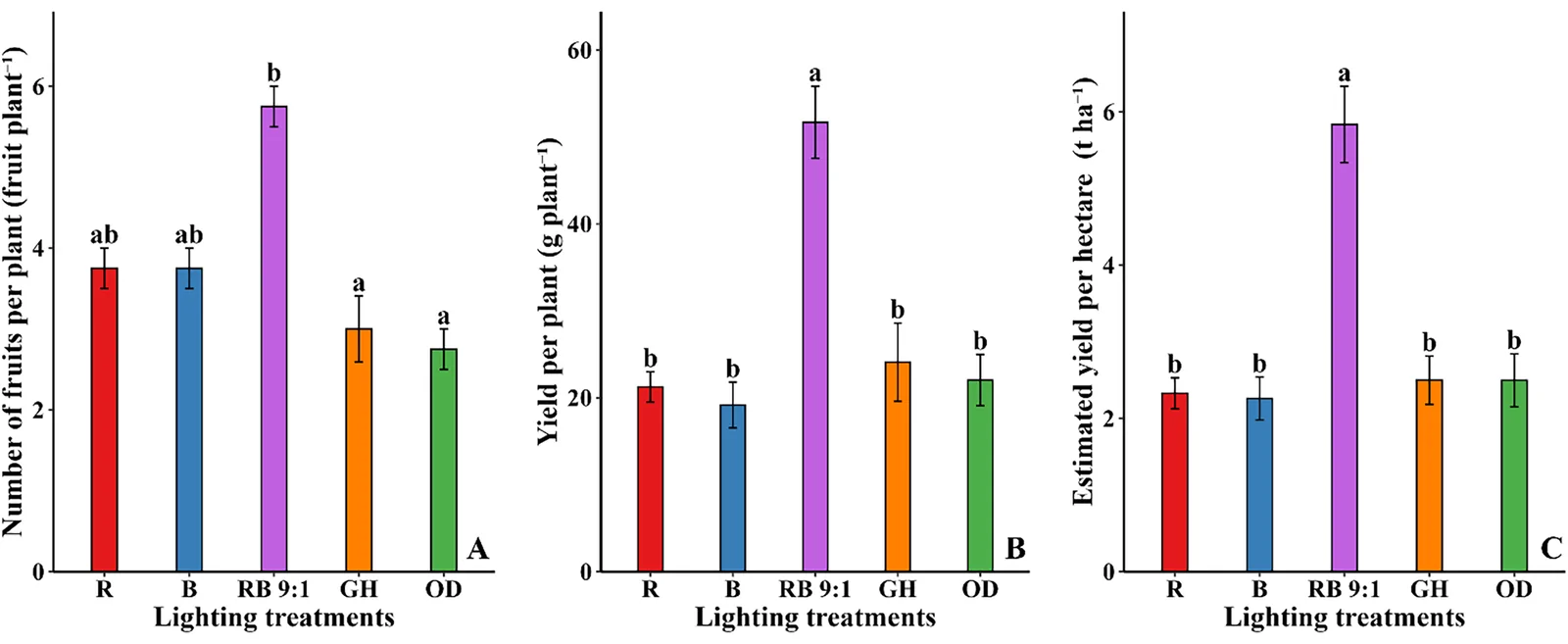
Yield components of okra (*A. esculentus* L.) plants grown under red (R), blue (B), and red-blue (RB 9:1) LED light, greenhouse (GH), and outdoor (OD) conditions. **(A)** Number of fruits per plant, **(B)** yield per plant, and **(C)** estimated yield per hectare. Bars represent mean values ± standard error (SE) (*n = 4*). Different letters indicate significant differences among treatments according to Tukey’s HSD test or Dunn’s *post hoc* test (*p< 0.05*).

Yield per plant (**Figure 12B**) and estimated yield per hectare (**Figure 12C**) were significantly increased under the RB 9:1 treatment. Over the 60-day experimental period, this treatment produced the highest yield per plant (51.69 ± 4.13 g plant^−1^) and estimated yield per hectare (5.84 ± 0.50 t ha^−1^), both more than twice the values recorded under the greenhouse (24.10 ± 4.49 g plant^−1^; 2.50 ± 0.32 t ha^−1^), outdoor (22.03 ± 2.94 g plant^−1^; 2.50 ± 0.35 t ha^−1^), R (21.26 ± 1.73 g plant^−1^; 2.33 ± 0.20 t ha^−1^), and B (19.18 ± 2.62 g plant^−1^; 2.26 ± 0.28 t ha^−1^) treatments. The latter four treatments showed similar values.

### 3.3 Effects of light treatments on fruit biochemical quality

Light treatments had a highly significant effect on DPPH radical inhibition (**Figure 13A**). The highest antioxidant activity was recorded under the RB 9:1 treatment (75.87 ± 1.26%), although it did not differ significantly from the R (75.55 ± 1.59%) and B (71.58 ± 1.68%) treatments. These three LED treatments exhibited significantly higher antioxidant activity than the greenhouse (55.04 ± 1.89%) and outdoor (54.95 ± 2.02%) treatments, which did not differ significantly from each other. No significant differences were observed in total phenolic content among the light treatments (**Figure 13B**), with values ranging from 15.44 ± 0.74 mg GAE g^−1^ DW under the greenhouse treatment to 19.55 ± 1.27 mg GAE g^−1^ DW under the B treatment.

**Figure 13:**
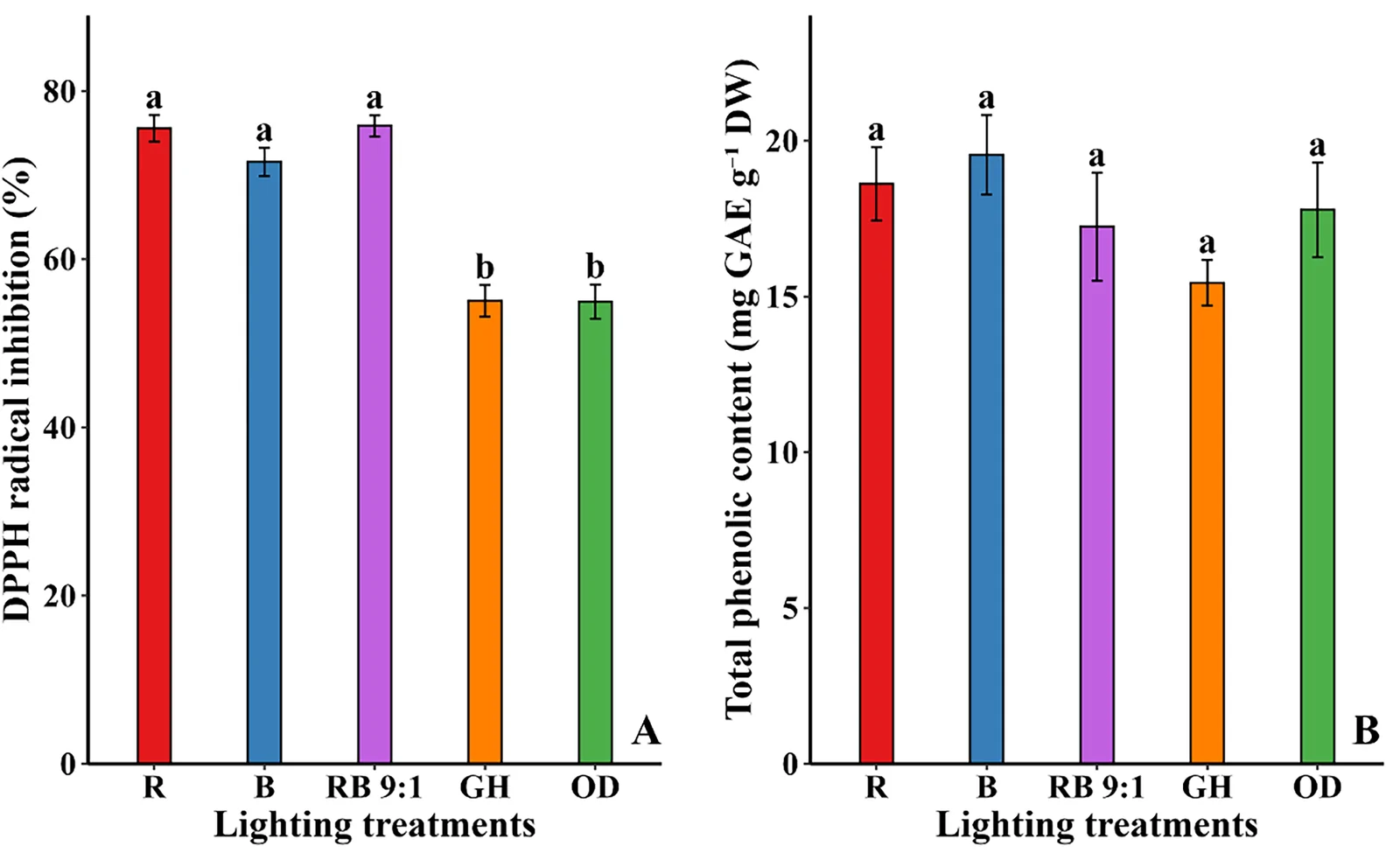
Antioxidant activity and total phenolic content of okra (*A. esculentus* L.) fruits grown under red (R), blue (B), and red-blue (RB 9:1) LED light, greenhouse (GH), and outdoor (OD) conditions. **(A)** DPPH radical inhibition and **(B)** total phenolic content. Bars represent mean values ± standard error (SE) (*n = 4*). Different letters indicate significant differences among treatments according to Tukey’s HSD test or Dunn’s *post hoc* test (*p< 0.05*).

Light treatments significantly affected the concentrations of certain macronutrients in okra fruits, particularly calcium, potassium, and sulfur, whereas phosphorus concentration was not significantly influenced (**Table 3**). Calcium concentration was significantly affected by the growing environment. RB 9:1 showed the highest numerical calcium concentration (1.75 ± 0.07% DW), although it did not differ significantly from the B treatment (1.41 ± 0.22% DW). The greenhouse treatment exhibited the lowest calcium concentration (0.73 ± 0.07% DW). In contrast, no significant differences were observed in phosphorus concentration among the growing environments, although the highest numerical mean was recorded under outdoor conditions (1.72 ± 0.06% DW).

**Table 3:** Macronutrient composition of okra (*A. esculentus* L.) fruits grown under red (R), blue (B), and red-blue (RB 9:1) LED light, greenhouse (GH), and outdoor (OD) conditions.

	Macronutrients (%·DW)			
Lighting treatments	Calcium (%·DW)	Phosphorus (%·DW)	Potassium (%·DW)	Sulfur (%·DW)
R	0.9 ± 0.13 bc	1.27 ± 0.09 a	11.47 ± 0.19 a	1.01 ± 0.06 c
B	1.41 ± 0.22 ab	1.45 ± 0.13 a	10.52 ± 0.38 ab	1.25 ± 0.08 b
RB 9:1	1.75 ± 0.07 a	1.36 ± 0.13 a	11.79 ± 0.12 a	1.87 ± 0.05 a
GH	0.73 ± 0.07 c	1.26 ± 0.12 a	8.97 ± 0.45 c	1.19 ± 0.02 bc
OD	0.95 ± 0.05 bc	1.72 ± 0.06 a	9.37 ± 0.31 bc	0.75 ± 0.04 d

Mean values ± standard error (SE) (*n = 4*). Different letters indicate significant differences among treatments according to Tukey’s HSD test (*p< 0.05*).

Potassium concentration was also significantly influenced by the growing environment. RB 9:1 showed the highest numerical potassium concentration (11.79 ± 0.12% DW), which did not differ significantly from the R (11.47 ± 0.19% DW) and B (10.52 ± 0.38% DW) treatments, but was significantly higher than the greenhouse (8.97 ± 0.45% DW) and outdoor (9.37 ± 0.31% DW) treatments. Similarly, sulfur concentration was significantly affected by the growing environment. RB 9:1 showed the highest sulfur concentration (1.87 ± 0.05% DW) and was significantly higher than all other environments, whereas outdoor showed the lowest concentration (0.75 ± 0.04% DW).

Light treatments significantly affected the concentrations of chlorine, copper, iron, and zinc in okra fruits, whereas molybdenum concentration was not significantly influenced (**Table 4**). Chlorine concentration was highest under the outdoor, RB 9:1, and greenhouse treatments, which did not differ significantly from one another, whereas the R and B treatments exhibited significantly lower concentrations. Copper concentration was highest under the RB 9:1, outdoor, and B treatments, which did not differ significantly from one another, while the greenhouse treatment recorded the lowest concentration. The R treatment showed an intermediate value and did not differ significantly from either group.

**Table 4:** Micronutrient composition of okra (*A. esculentus* L.) fruits grown under red (R), blue (B), and red-blue (RB 9:1) LED light, greenhouse (GH), and outdoor (OD) conditions.

	Micronutrients (mg·kg^−1^·DW)				
Lighting treatments	Chlorine (mg·kg^−1^·DW)	Copper (mg·kg^−1^·DW)	Iron (mg·kg^−1^·DW)	Molybdenum (mg·kg^−1^·DW)	Zinc (mg·kg^−1^·DW)
R	17905 ± 386.75 b	37.5 ± 3.23 ab	52.5 ± 4.79 d	10.97 ± 0.29 a	177.5 ± 10.31 a
B	18075 ± 371.4 b	41.5 ± 4.05 a	62.5 ± 4.79 cd	11.79 ± 0.36 a	137.5 ± 4.79 b
RB 9:1	23512.5 ± 435.5 a	45 ± 2.89 a	97.5 ± 8.54 ab	11.12 ± 0.28 a	195 ± 6.45 a
GH	23477.5 ± 601 a	25.75 ± 1.55 b	80 ± 4.08 bc	10.64 ± 0.51 a	130 ± 7.07 bc
OD	25415 ± 798.65 a	42.5 ± 3.23 a	115 ± 6.45 a	10.57 ± 0.46 a	100 ± 9.13 c

Mean values ± standard error (SE) (*n = 4*). Different letters indicate significant differences among treatments according to Tukey’s HSD test (*p< 0.05*).

Iron concentration followed a different pattern, reaching its highest level under outdoor conditions, although it did not differ significantly from the RB 9:1 treatment. In contrast, the R treatment exhibited the lowest iron concentration, whereas the B and greenhouse treatments showed intermediate concentrations. Molybdenum concentration was not significantly affected by the light treatments. Finally, zinc concentration was highest under the RB 9:1 and R treatments, which did not differ significantly from each other, whereas outdoor-grown fruits exhibited the lowest concentration, with the B and greenhouse treatments showing intermediate values.

Light treatments significantly affected the concentrations of several trace elements in okra fruits, including rubidium, silicon, and strontium, whereas chromium concentration was not significantly influenced (**Table 5**). Although chromium concentration did not differ significantly among treatments, the highest numerical mean was recorded under the RB 9:1 treatment, followed by the outdoor and greenhouse treatments. The highest measured rubidium concentration was observed under R treatment, which was significantly higher than those under greenhouse and outdoor conditions. The RB 9:1 treatment showed an intermediate concentration that did not differ significantly from R or B, whereas B did not differ significantly from greenhouse or outdoor conditions.

**Table 5:** Trace elements composition of okra (*A. esculentus* L.) fruits grown under red (R), blue (B), and red-blue (RB 9:1) LED light, greenhouse (GH), and outdoor (OD) conditions.

	Trace elements (mg·kg^−1^·DW)			
Lighting treatments	Chromium (mg·kg^−1^·DW)	Rubidium (mg·kg^−1^·DW)	Silicon (mg·kg^−1^·DW)	Strontium (mg·kg^−1^·DW)
R	35 ± 5 a	140 ± 4.08 a	4620 ± 313.34 ab	11.08 ± 0.69 bc
B	37.5 ± 4.79 a	112.5 ± 4.79 bc	3670 ± 401.58 b	10.46 ± 0.33 c
RB 9:1	50 ± 4.08 a	127.5 ± 4.79 ab	5170 ± 106.14 a	13.06 ± 0.43 ab
GH	42.5 ± 4.79 a	102.5 ± 4.79 c	5020 ± 300.97 ab	11.54 ± 0.22 bc
OD	45 ± 6.45 a	107.5 ± 2.5 c	4570 ± 405.94 ab	13.55 ± 0.49 a

Mean values ± standard error (SE) (*n = 4*). Different letters indicate significant differences among treatments according to Tukey’s HSD test (*p< 0.05*).

A different pattern was observed for silicon, with RB 9:1 showing the highest numerical concentration. However, it did not differ significantly from the R, greenhouse, or outdoor treatments, whereas the B treatment showed the lowest numerical concentration. Similarly, strontium concentration was highest under outdoor conditions, although it did not differ significantly from the RB 9:1 treatment. The R and greenhouse treatments showed intermediate concentrations, while the B treatment had the lowest numerical concentration.

## 4 Discussions

### 4.1 Flowering responses to light treatments

Flowering is a critical stage in the plant developmental cycle, determining reproduction and productivity. It results from the interaction between environmental signals, particularly photoperiod and light spectral quality, and internal hormonal regulation (Lin, 2000). In okra, a quantitative short-day plant, these factors play a central role in the floral transition and productivity (Maduagwuna et al., 2019; Yao et al., 2025).

Our results show a highly significant effect of the growing environment on the day of first floral bud initiation and on the day of first flower opening (**Figure 7**). The RB 9:1 treatment induced the earliest floral initiation, significantly faster than under the greenhouse environment and the B treatment. A similar pattern was observed for first flower opening, with RB 9:1 showing the earliest anthesis. These results indicate that RB 9:1 was associated with earlier flowering than the monochromatic blue treatment under the tested LED conditions. However, comparisons with the greenhouse and outdoor environments reflect the combined effects of spectral composition, photoperiod, PPFD, DLI, relative humidity, and other environmental factors; therefore, the specific contribution of spectral quality to the observed flowering responses cannot be isolated. Similar effects of light quality on flowering have been reported in other short-day plants (Amaki and Kunii, 2015).

The delay observed under the greenhouse environment, characterized by a lower mean PPFD than the outdoor environment, is consistent with the findings of Dada and Adejumo (Dada and Adejumo, 2015), who reported that a marked reduction in light intensity can delay flowering in okra. However, because PPFD, photoperiod, DLI, and other environmental conditions differed among the growing environments in the present study, the specific contribution of light intensity to the observed differences cannot be isolated. Furthermore, as a quantitative short-day plant, okra flowers earlier under short photoperiods, which may have contributed to the earlier flowering observed under the 10-h photoperiod compared with the natural photoperiod of approximately 11.4 hours. This result is consistent with those reported in four okra varieties, in which a 12 h photoperiod delayed floral initiation and flowering compared with a 10 h photoperiod (Maduagwuna et al., 2019). The fact that the outdoor environment, despite having the same natural photoperiod as the greenhouse environment, showed earlier flowering may suggest a contribution of light intensity to flowering responses, although the specific contribution of PPFD cannot be separated from other environmental differences between the two environments.

Relative humidity may also influence flowering responses. Mortensen (Mortensen, 1986), reported that increasing relative humidity reduced the time to flowering in several ornamental species. In the present study, however, earlier flowering was observed under LED conditions despite the lower relative humidity (47%) compared with outdoor (72%) and greenhouse (74%) conditions. Thus, the lower relative humidity alone is unlikely to explain the earlier flowering observed under LED conditions, although its contribution cannot be completely excluded.

In this study, the earlier flowering observed under red/blue 9:1 and red light compared with blue light may be associated with differences in spectral composition. However, the contribution of spectral quality cannot be considered independently of photoperiod and PPFD when comparing these treatments with the greenhouse and outdoor environments. Red and blue light are primarily perceived by phytochromes and cryptochromes, respectively, which can regulate flowering through signaling pathways involving CONSTANS (CO) and FLOWERING LOCUS T (FT) (Cho et al., 2017; Putterill et al., 2004; Shibuya and Kanayama, 2014). The earlier flowering observed under RB 9:1 and red light may therefore be associated with differential regulation of these light-signaling pathways. However, this interpretation remains hypothetical because no measurements of CO, FT, phytochrome B (PHYB), cryptochrome 2 (CRY2), or other flowering-related genes were performed in okra. Similar behavior has been reported in *Kalanchoe blossfeldiana*, a typical short-day plant, in which red light significantly accelerates flowering while blue light delays it (Amaki and Kunii, 2015). The red/blue combination (9:1) may therefore provide a spectral condition favorable to earlier flowering, consistent with observations reported in other ornamental species (Heo et al., 2003; Wojciechowska et al., 2019). These proposed photoreceptor-mediated mechanisms should be considered hypothesis-generating rather than demonstrated mechanisms in the present study. Further molecular studies in okra, including analyses of flowering-related gene expression, would be required to determine whether these pathways contribute to the observed flowering responses.

Finally, the node bearing the first floral bud was not significantly affected by light treatments, with floral buds consistently initiated at the fifth or sixth node regardless of treatment (**Figure 7C**). This result suggests that the morphological position of floral initiation remains stable under short photoperiods, in contrast to what has been observed under longer photoperiods or night interruption, where light quality can influence nodal position in okra (Hamamoto and Yamazaki, 2009; Yao et al., 2025). Photoperiod may therefore contribute to the regulation of this morphological trait, although its relative contribution compared with spectral quality cannot be determined from the present experimental design (Nwoke, 1983).

### 4.2 Fruit morphology and yield responses to light treatments

Light spectral quality, intensity, and photoperiod strongly influence morphological traits, fruit biomass, and crop yield (Ouzounis et al., 2015; Roosta et al., 2024). In the present study, okra plants grown under RB 9:1 showed the highest numerical fruit length and fresh fruit mass, while this treatment also produced the highest number of fruits per plant, yield per plant, and estimated yield per hectare over the 60-day experimental period (**Figures 9**, **12**). These results are consistent with those of Díaz-Galián et al (Díaz-Galián et al., 2021), who showed that a combination of red and blue light significantly increases strawberry production compared with plants grown under natural greenhouse light. Similarly, a red/blue ratio of 3:1 improved fruit mass, fruit length, and early yield in the ‘Albion’ strawberry cultivar compared with red, blue, or white+yellow spectra (Roosta et al., 2024). Furthermore, combined red/blue lighting increased the number of harvested fruits while shortening the fruit ripening period (Hidaka et al., 2016). Collectively, these findings highlight that optimizing spectral quality through an appropriate combination of wavelengths may contribute to improved plant growth, reproductive development, and cumulative fruit production.

The higher fruit yield observed under RB 9:1 may also be interpreted through a hormonal lens. In radish, red light has been shown to markedly increase gibberellin (GA) content in the aboveground parts, enhancing their sink activity, whereas blue light stimulates the synthesis of cytokinins and indole-3-acetic acid (IAA) in the hypocotyl (Kara et al., 1997). Cytokinins are known to promote cell division, pedicel growth, and the transport of water, nutrients, and assimilates toward the fruit, thereby determining final fruit size (Matsuo et al., 2012). A combined red/blue spectrum may therefore allow a more balanced hormonal profile, with red light supporting gibberellin-mediated assimilate demand and blue light sustaining cytokinin-driven transport toward reproductive organs, together improving assimilate allocation and consequently increasing fruit size and mass. A comparable mechanism may account for the improved fruit mass, fruit number, and higher fruit yield observed under RB 9:1 in the present study. This interpretation is further supported by evidence in grape, where IAA content in flower buds under combined red/blue light was higher than under natural sunlight throughout floral bud differentiation (Liu et al., 2023), indicating that the red/blue combination itself, beyond the individual contribution of each wavelength, can enhance auxin-related signaling relative to natural light conditions. Importantly, although RB 9:1 induced earlier flowering, this treatment produced a greater number of fruits over the 60-day experimental period. The 60-day experimental period was sufficient to capture the fruit production resulting from the floral buds formed during the experiment, as no new floral buds were observed at the end of the experiment in any of the lighting treatments, and the last fruits had reached the harvest stage. Therefore, the higher fruit production observed under RB 9:1 reflects its earlier reproductive development and greater fruit production within the experimental period, rather than an ongoing flowering process in any of the treatments at the experimental endpoint. However, hormonal studies measuring gibberellin, cytokinin, and IAA levels during the reproductive phase are needed in okra to directly confirm these interpretations.

Estimated yield per hectare was numerically higher under red light than under blue light, although the difference was not statistically significant. Comparable observations have been reported in the ‘Parous’ strawberry cultivar, where red light led to a higher yield than blue light (Roosta et al., 2024). Similarly, Cho et al. (Cho et al., 2019) showed that red light increases blueberry fruit mass and size compared with blue light. However, these effects of monochromatic spectra appear to be strongly species- and cultivar-dependent (H. Y. Cho et al., 2019; Roosta et al., 2024).

In our study, the higher fresh mass but slightly lower dry mass of okra fruits under red/blue light compared with the greenhouse control, without a significant difference, could be explained by a higher water content in fruits under this treatment. Furthermore, the larger diameter observed under greenhouse conditions could be related to the slightly longer photoperiod. Indeed, a natural photoperiod of approximately 11.5 hours has been associated with an increase in average okra fruit diameter compared with a 10-hour photoperiod (Maduagwuna et al., 2019).

### 4.3 Fruit biochemical quality responses to light treatments

As a defense strategy against biotic and abiotic stress, plants synthesize antioxidant compounds such as phenolics to shield the photosynthetic machinery from oxidative damage (Lattanzio et al., 2006). Light intensity modulates antioxidant activity as well as total phenolic content, as observed in basil and lettuce (Sutulienė et al., 2022). In this study, LED treatments with high light intensity significantly improved antioxidant activity in okra fruits compared with the greenhouse and outdoor controls; in contrast, total phenolic content, although numerically higher under these treatments, did not differ significantly from the controls (**Figure 13**). Light quality is also among the environmental parameters that trigger the biosynthesis of these compounds (Kopsell et al., 2004). These wavelengths blue, red, and far-red can act on phenolic biosynthesis either directly, by switching on the expression of key biosynthetic enzymes, or indirectly, by increasing the pool of shikimic acid, a precursor from which phenolic compounds are derived (Giménez et al., 2021; Loi et al., 2021).

For instance, some researchers have reported that the use of red and blue light, whether as single or combined spectra, improved the antioxidant properties of several vegetables, such as wheat and tomato, compared with white light sources (Dong et al., 2014; Hee-Sun Kook, 2013). A red/blue ratio of 9:1 was more effective at enhancing antioxidant activity in green basil, garlic chives, and borage than red/blue ratios of 5:1 and 2:1 (Bantis, 2021). Our results corroborate those of Naznin et al (Naznin et al., 2016), who found higher antioxidant activity in coriander plants grown under combined red and blue LEDs compared with those grown under red LEDs alone. In contrast, red light promoted greater antioxidant accumulation in young pea seedlings than blue light (Wu et al., 2007), underscoring that the antioxidant response to light spectrum remains strongly dependent on species and tissue type.

Regarding total phenolic content, the blue-light treatment showed the highest numerical value, although no significant differences were observed among the growing environments (**Figure 13B**). This numerical trend may be consistent with previous reports indicating that blue light can influence phenolic compound accumulation in several plant species. For example, blue LED lighting has been associated with higher phenolic compound contents in Chinese kale and red amaranth (*Amaranthus tricolor* L.) compared with white (Qian et al., 2016; Toscano et al., 2021). Similarly, *Brassica carinata* microgreens exposed to monochromatic blue light produced higher phenolic compound contents than those exposed to red light or a 50% red/50% blue mixture (Maina et al., 2021), while increasing the proportion of blue light has been associated with greater phenolic accumulation in lamb’s lettuce (Długosz-Grochowska et al., 2016). These responses have been proposed to involve blue-light photoreceptors such as cryptochromes, which can regulate metabolic pathways associated with secondary metabolite biosynthesis (Kopsell and Sams, 2013). However, this numerical difference was not statistically significant and should therefore be interpreted as a trend rather than a treatment effect.

Furthermore, nutrient availability plays a key role in plant growth and development. Recent studies have shown that LED lighting, particularly red and blue wavelengths, modulates nutrient uptake, transport, and assimilation, thereby contributing to improved crop productivity and quality in controlled environments (Bouain et al., 2019; Landi et al., 2020; Trivellini et al., 2023). More specifically, mineral accumulation in leaves and fruits can be altered by variations in light properties, including intensity, duration, and spectral quality (Xu et al., 2021). Our results revealed element-specific responses to the different growing environments. The RB 9:1 treatment showed the highest measured concentrations of calcium (Ca), potassium (K), sulfur (S), copper (Cu), silicon (Si), and zinc (Zn), although these values were not always significantly different from those observed under other growing environments (**Tables 3**-**5**). In contrast, the highest numerical concentration of rubidium (Rb) was observed under the red treatment, while iron (Fe), chlorine (Cl), and strontium (Sr) showed their highest numerical concentrations under outdoor conditions. Phosphorus (P), chromium (Cr), and molybdenum (Mo) did not differ significantly among the growing environments. These element-specific responses are consistent with recent controlled-environment research showing that different light spectra can produce contrasting effects depending on the crop trait evaluated. For example, (Lee et al., 2025) reported that different LED spectra produced distinct responses in biomass production, plant quality, and physiological performance of Korean thistle, indicating that no single spectrum was universally optimal across all evaluated traits.

Regarding the elements unaffected by light quality, a similar pattern has been reported in tomato (*Solanum lycopersicum*), where P concentration was not affected by natural light or by high-pressure sodium (HPS) lamps under different intensities and photoperiods (Tremblay et al., 1987). Likewise, different red:green:blue, red:blue, or red:far-red:blue light quality ratios, applied under varying light intensities, did not affect P, Cu, or manganese (Mn) concentrations in Brassicaceae microgreens (Gerovac et al., 2016). Red light has nevertheless been shown to promote phosphorus uptake and accumulation through a phytochrome B (PHYB)–LONG HYPOCOTYL 5 (HY5) signaling pathway (Sakuraba and Yanagisawa, 2018). However, the absence of a significant difference in P concentration in the present study does not allow us to determine whether a similar PHYB–HY5 pathway operates in okra. Thus, its potential involvement in the regulation of phosphorus accumulation in okra fruits remains hypothetical and requires further molecular investigation.

Furthermore, the highest numerical Fe, Cl, and Sr concentrations observed under outdoor conditions may be associated with the broader solar spectrum, including green and far-red spectral bands, which have been reported to influence nutrient uptake and accumulation in plants (Pinho et al., 2017). This difference may also be associated with the longer photoperiod under outdoor conditions compared with artificial lighting. In young tomato plants, a low light intensity combined with a long photoperiod resulted in higher uptake of major ions than natural light or high-intensity, short-photoperiod treatments (Tremblay et al., 1987). This possibility warrants further investigation in okra, particularly because photoperiod can also influence flowering responses in short-day plants.

Regarding the elements that were more abundant under red/blue and red treatments, this trend is consistent with numerous studies reporting that a high red/blue ratio, or red light alone, promotes the uptake of macro- and micronutrients (N, P, K, Mg, Ca, Fe, Zn, Se) in various species such as lettuce, basil, celery, cucumber, and tomato (De Wit et al., 2016; Pennisi et al., 2019, 2019; W. Wang et al., 2021; Xu et al., 2021), including in okra leaves themselves (Degni et al., 2021). This is consistent with the higher numerical concentrations of calcium, potassium, sulfur, copper, and zinc observed under red/blue (9:1) in the present study, as well as the higher numerical rubidium concentration under red light. These findings suggest that red-enriched light may be associated with differences in mineral accumulation in okra fruits under the tested conditions. Blue light may nevertheless attenuate some of the effects associated with red-light-driven nutrient accumulation (Miao et al., 2019). Such responses have been associated with the activity of phototropins, blue-light photoreceptors involved in the regulation of ion transport (Xu et al., 2021). However, this proposed mechanism was not investigated in the present study and therefore remains hypothetical.

Finally, like the blue treatment, the greenhouse treatment was also less effective for mineral accumulation in okra fruits, which could be explained by the lower light intensity under this condition. The lower relative humidity recorded under LED lighting (47%) compared with greenhouse (74%) and outdoor (72%) conditions may also have contributed to differences in plant physiological responses. Previous studies have shown that relative humidity can affect dry biomass, shoot growth, leaf development, and flowering, although the magnitude and direction of these effects are species-dependent (Mortensen, 1986). Ambient relative humidity can influence stomatal functioning and the balance between CO_2_ uptake and water loss, particularly through its effects on plant water relations (Fanourakis et al., 2020). However, because relative humidity differed inherently among the growing environments, its contribution to the observed responses could not be separated from the effects of light spectrum, photoperiod, and PPFD in the present study. These observations are consistent with the findings of Zhou et al (Zhou et al., 2019), who showed that N, P, and K uptake rates were significantly reduced in lettuce grown under low light intensity. However, increasing PPFD from 105 to 315 µmol·m^−2^·s^−1^ decreased the concentrations of certain macro- and micronutrients in some Brassica species and ornamental crops, but not in others (Craver et al., 2018; Gerovac et al., 2016), highlighting that the relationship between light intensity and mineral accumulation is not always linear or generalizable across species. The mechanisms underlying light-regulated nutrient uptake therefore remain to be elucidated in okra. Although PHYB/HY5-related signaling and phototropin activity may provide possible mechanistic explanations, these pathways were not measured in the present study. Further studies incorporating molecular and physiological measurements would therefore be needed to test these hypotheses.

## 5 Conclusions

The present study showed that okra responses varied according to the light environment and the trait evaluated. The red/blue (9:1) LED treatment promoted earlier reproductive development and showed the highest yield per plant and estimated yield per hectare over the 60-day experimental period. It also showed the highest measured concentrations of selected elements, including calcium, potassium, sulfur, copper, silicon, and zinc, although these responses were not statistically different from all other growing environments for each element. In contrast, outdoor conditions showed the highest numerical concentrations of iron, chlorine, and strontium, while red light showed the highest numerical rubidium concentration. For biochemical quality, RB 9:1 showed the highest numerical antioxidant activity, although it did not differ significantly from the red and blue treatments, while all LED treatments showed significantly higher antioxidant activity than the greenhouse and outdoor conditions. Blue light showed the highest numerical total phenolic content, although no significant differences were observed among growing environments.

Overall, the results indicate that okra responses to the tested light environments were trait- and element-specific. The red/blue (9:1) spectrum therefore represents a promising lighting condition for promoting earlier reproductive development and fruit production under the specific experimental conditions, cultivar, planting density, and 60-day cultivation period tested in this study. However, comparisons between LED, greenhouse, and outdoor environments were influenced by differences in photoperiod, daily light integral, relative humidity, and irrigation frequency. In addition, estimated yield per hectare represents an extrapolation from potted plants, and energy consumption and economic performance were not evaluated. Only one red/blue ratio (9:1) was evaluated in the present study. Further studies could explore different red-to-blue ratios and evaluate their agronomic, energy, and economic performance across different cultivars and controlled-environment conditions to identify spectral strategies that balance flowering, fruit production, fruit quality, and lighting efficiency.

## Data Availability

The raw data supporting the conclusions of this article will be made available by the authors, without undue reservation.
